# Long non-coding RNA GRASLND links melanoma differentiation and interferon-gamma response

**DOI:** 10.3389/fmolb.2024.1471100

**Published:** 2024-09-27

**Authors:** Kim Denise Fischer, Shashank Tiwari, Beatrice Thier, Lin Christina Qiu, Tzu-Chen Lin, Annette Paschen, Jochen Imig

**Affiliations:** ^1^ Chemical Genomics Centre, Max Planck Institute of Molecular Physiology, Dortmund, Germany; ^2^ Faculty of Chemistry and Chemical Biology, Technical University of Dortmund, Dortmund, Germany; ^3^ Department of Dermatology, University Hospital Essen, University Duisburg-Essen, Essen, Germany

**Keywords:** melanoma, lncRNA, phenotypic switch, differentiation, IFNγ response

## Abstract

Melanoma is a highly malignant tumor, that stands as the most lethal form of skin cancer and is characterized by notable phenotypic plasticity and intratumoral heterogeneity. Melanoma plasticity is involved in tumor growth, metastasis and therapy resistance. Long non-coding RNAs (lncRNAs) could influence plasticity due to their regulatory function. However, their role and mode of action are poorly studied. Here, we show a relevance of lncRNA GRASLND in melanoma differentiation and IFNγ signaling. GRASLND knockdown revealed switching of differentiated, melanocytic melanoma cells towards a dedifferentiated, slow-proliferating and highly-invasive cell state. Interestingly, GRASLND is overexpressed in differentiated melanomas and associated with poor prognosis. Accordingly, we found GRASLND expressed in immunological “cold” tumors and it negatively correlates with gene signatures of immune response activation. In line, silencing of GRASLND under IFNγ enhanced the expression of IFNγ-stimulated genes, including HLA-I antigen presentation, demonstrating suppressive activity of GRASLND on IFNγ signaling. Our findings demonstrate that in differentiated melanomas elevated expression of GRASLND interferes with anti-tumor effects of IFNγ, suggesting a role of GRASLND in tumor immune evasion.

## 1 Introduction

Melanoma is a profoundly aggressive tumor with a substantial mortality rate, notable for its high metastatic nature (Cummins et al., 2006). Despite the revolutionary impact of targeted and immunotherapies on metastatic melanoma treatment, the majority of patients face dismal clinical outcomes as therapy resistance becomes a prevalent challenge (Rizos et al., 2014; Sharma et al., 2017). Resistance development and metastatic dissemination is linked to intratumoral heterogeneity and cellular plasticity allowing the adaptation to environmental changes (Meacham and Morrison, 2013; Rambow et al., 2019). The phenomenon of phenotype switching describes this intricate cellular plasticity, in which melanoma cells undergo reversible transcriptional reprogramming that relies on distinct expression levels of microphthalmia-associated transcription factor (MITF) (Hoek et al., 2006). MITF regulates melanocytic lineage-specific gene expression controlling melanoma development and differentiation (Steingrímsson et al., 2004). The distinct phenotypes were initially described as a differentiated, melanocytic and ‘epithelial-like’ MITFhigh cell state, that is highly proliferative and as a dedifferentiated, ‘mesenchymal-like’ and invasive, MITFlow cell state (Hoek et al., 2006; Tirosh et al., 2016). Moreover, a neural-crest-like and an intermediate cell state have been reported, with the latter exhibiting a transcriptome characterized by gene regulatory networks from both melanocytic and mesenchymal-like cell states (Wouters et al., 2020). In response to microenvironmental cues, melanoma cells are capable to switch between these cell states. Phenotype switching to a dedifferentiated cell state, a process showing similarities with the epithelial to mesenchymal transition (EMT), has been described to promote melanoma progression and metastasis (Alonso et al., 2007; Li et al., 2015), and is associated with resistance to targeted or immunotherapies (Konieczkowski et al., 2014; Müller et al., 2014; Mehta et al., 2018; Lee et al., 2020). In this regard, long non-coding RNAs (lncRNAs) have recently gained increasing attention as key regulators of precisely these processes in melanomagenesis (Melixetian et al., 2022). Their investigation offers the potential to develop innovative strategies to address melanoma plasticity and counteract therapy resistance.

lncRNAs are RNA molecules with over 200 nucleotides that are not coding for proteins, but influence diverse cellular processes within the cell due to their regulatory properties. The remarkable functional diversity of lncRNAs comprises the modulation of gene expression on the epigenetic, transcriptional and post-transcriptional level in a tissue- and cell-type specific manner (Khalil and Coller, 2013). Hence, dysregulation of the lncRNA transcriptome exerts profound impacts on cellular function contributing to the development of diseases, most notably cancer (Huarte, 2015). Specifically in melanoma, lncRNAs are reported to induce phenotype switching and metastasis formation by miRNA sponging or epigenetic regulator functions (Luan et al., 2017; Luan et al., 2018; Wang et al., 2019; Sheng and Wei, 2020). In addition, several lncRNAs have been identified as potential contributors to resistance to targeted therapy applying BRAF and MEK inhibitors (Sanlorenzo et al., 2018; Galus et al., 2024), and chemotherapy with platinum compounds (Pan et al., 2019; An et al., 2020). The effectiveness of immunotherapy crucially depends on the ability of the immune system to recognize tumor cells. Immune-related lncRNA signatures that impact this process have already been documented using bioinformatic prediction and correlation models (Wang et al., 2021; Zhou et al., 2021). As of now, the only known lncRNA described for a direct influence on immune response in melanoma and a revealed mechanism for activating HLA-I antigen presentation is LIMIT (Li et al., 2021). Those recent findings highlight the pivotal involvement of lncRNAs in key aspects of melanogenesis and their clinical relevance. However, further research is required to elicit their exact molecular mechanisms.

In this study, we investigated the function of the primate-specific lncRNA RNF144A-AS1 and its potential contribution in melanoma progression. RNF144A-AS1 was firstly discovered as a critical regulator in chondrogenesis in mesenchymal stem cells (MSCs) by suppressing IFNγ signaling and was named after its function as Glycosaminoglycan Regulatory Associated Long Non-coding RNA (GRASLND) (Huynh et al., 2020). Previous studies have indicated that GRASLND serves as an unfavourable prognostic marker in several cancer types (Song et al., 2018; Hu et al., 2020; Chen et al., 2021; Liu et al., 2021; Rothzerg et al., 2021; Zhong et al., 2022), due to the promotion of cell proliferation, migration and invasion in glioma, bladder and gastric cancer (Wang et al., 2020; Ding et al., 2021; Tong et al., 2021; Li et al., 2021). A recent study suggested that GRASLND has pro-tumorigenic activity by enhancing YAP1 signaling in melanoma, thereby promoting its tumorigenicity (Yang et al., 2024).

Our findings revealed GRASLND’s cell state-dependent function as a suppressor of the invasive and dedifferentiated melanoma phenotype and demonstrated that this lncRNA is dominantly expressed in differentiated, melanocytic melanoma cells. It is upregulated in tumorous tissue compared to healthy skin and is associated with poor patient prognosis. We further identified GRASLND as a potential factor contributing to immune evasion by inhibiting the IFNγ signaling.

## 2 Materials and methods

### 2.1 Cell culture

Human melanoma cell lines 501-mel (RRID:CVCL_4633; kindly provided by Aifantis Lab, NYU), SK-MEL-239 (RRID:CVCL_6122, received from Memorial Sloan Kettering Cancer Center (MSK), SK-MEL-147 (RRID:CVCL_3876, received from MSK), A375 (RRID:CVCL_0132, ATCC), C8161 (RRID:CVCL_6813; kindly provided by Mary J. C. Hendrix, Department of Biology, Shepherd University, Shepherdstown, WV, United States and West Virginia University Research Corporation) and lentiviral production cell line Lenti-X 293T cells (RRID:CVCL_4401; Takara) were cultured in Dulbecco’s Modified Eagle Medium (DMEM) medium, high glucose with pyruvate supplemented with 10% (v/v) fetal bovine serum (FBS) and 1% (v/v) penicillin/streptomycin. The previously described melanoma cell lines Ma-Mel-86a (RRID:CVCL_A221), Ma-Mel-86c (RRID:CVCL_C7TP) and Ma-Mel-61a (RRID:CVCL_C291) (Zhao et al., 2016; Stupia et al., 2023) were cultured in RPMI1640 medium supplemented with 10% (v/v) fetal bovine serum (FBS) and 1% (v/v) penicillin/streptomycin. Melanoma cell line WM1361a cells were cultured in medium containing 80% (v/v) MCDB153 with L-glutamine and 28 mM HEPES, 20% (v/v) Leibovitz L-15, supplemented with NaHCO_3_ (1.2 g/L), 2% heat inactivated FBS, CaCl_2_ (1.68 mM), insulin from bovine pancreas (5 μg/mL) and 1% (v/v) penicillin/streptomycin. All cells were grown in a humidified incubator at 5% CO_2_ and 37°C. All cell lines were verified for the absence of *Mycoplasma* by using the LookOut *Mycoplasma* PCR Detection Kit (Sigma Aldrich).

### 2.2 Total RNA extraction and RT-qPCR

Total RNA was isolated using TRIzol™ Reagent according to the manufacturer’s guidelines and using GlycoBlue™ Co-precipitant (Thermo Fisher). Subsequent reverse transcription was performed using High-Capacity cDNA Reverse Transcription Kit (Thermo Fisher) following the manufacturer’s protocol. cDNA was stored at −20°C until further use. Real-time quantitative PCR was performed using GoTaq^®^ qPCR Master Mix (Promega) following the user guide. GAPDH or HPRT served as reference genes. The primers used are listed in Supplementary Table S1.

### 2.3 Western blot

Proteins were extracted from cells using RIPA buffer (Cold Spring Harbor Protocol 2017) supplied with 1 × Complete Protease Inhibitor Cocktail (Roche) and quantified with Pierce™ BCA Protein Assay Kit (Thermo Scientific) according to the manufacturer’s instructions. 20–30 μg protein was separated on a 10% SDS-polyacrylamide gel and electroblotted onto a PVDF membrane. The membrane was blocked with 5% non-fat dry milk for 30 min at room temperature. Incubation with primary antibody was performed overnight at 4°C using the following antibodies: anti-human rabbit antibodies anti-AXL (clone C89E7, Cell Signaling), anti-PARP (Cell Signaling), anti-STAT3 (clone D3Z2G, Cell Signaling), anti-PKR (Proteintech) and anti-β-actin (clone 13E5, Cell Signaling). Anti-human mouse antibodies used: anti-MITF (clone C5, Sigma-Aldrich), anti-MelanA (clone M2-7C10, Santa Cruz Biotechnology, Inc.), anti-GAPDH (clone 1E6D9, Proteintech), anti-Vinculin (clone hVIN-1, Sigma Aldrich). Subsequently, the membrane was washed and incubated with appropriate secondary HRP-conjugated antibodies with (Sigma Aldrich). Clarity™ Western ECL Substrate (Bio-Rad) was used for visualization and detection. GAPDH, Vinculin or β-actin served as loading controls. Band intensities were quantified using the image processing software ImageJ. The steps for quantification include the adjustment of the contrast to eliminate non-specific signals, followed by drawing of rectangular regions around each band to be quantified. Band intensities were measured using the “Gel” function and normalized to the loading control bands. The normalized intensities values from three independent biological replicates were used for statistical analysis. Uncropped images of all western blots are shown in Supplementary Figure S3.

### 2.4 Real-time live-cell imaging and analysis

Stable shRNA knockdown 501-mel cells were seeded 1 day prior to induction at 1.5 × 10^3^ cells/well in a 96-well plate. Doxycycline was added to the cells at a final concentration of 2 μg/mL. Control cells were left untreated. Cellular growth behavior was monitored for 5 days using the IncuCyte S3 System. Cell confluency was analyzed as a measure of cell growth using the IncuCyte Software 2019B Rev2.

### 2.5 Transwell invasion assay

Cell invasiveness of stable shRNA knockdown 501-mel cells was determined using FluoroBlok™ 24-well Transwell inserts (Corning, 8.0 µm colored PET Membrane). The membrane was pre-coated with 100 µL matrigel (Corning) at a final concentration of 300 μg/mL in coating buffer solution (0.01 M Tris-HCl pH 8.0, 0.7% NaCl) by incubation at 37°C for 2 h. Afterwards, residual matrigel was aspirated. Cells were starved overnight in serum-free media, detached and resuspended in serum-free medium. For each condition, 5 × 10^4^ cells were added in 300 µL of serum-free medium onto the membrane and settled for 10 min 700 μL medium with 10% FBS and 1 µM lysophosphatidic acid was filled into the lower chamber. After the incubation at 37°C for 48 h, the transmigrated cells were post-stained by incubation of the inserts in Calcein AM (Abcam) diluted to 2 μg/mL in HBSS at 37°C for 10 min. The stained cells were then imaged with a DeltaVision Elite Imaging System (GE Healthcare), which was based on an Olympus IX-71 stand and operated with softWoRx 7.2.0 software. For each insert, 10 images were taken using a × 10 objective for a complete coverage of the whole membrane. For each independent experiment, two inserts were used per condition. 501-mel cells harboring control (lacZ) or GRASLND shRNAs were treated with 2 μg/mL doxycycline for 72 h. Quantification of the invading cells was performed according to Hanniford et al. (2020) with slight changes. The following automated macro in ImageJ (FIJI) was used for image processing, coloring and cell counting.

#### 2.5.1 Processing code

macro “Batch Convert to Binary” {

dir = getDirectory (“Choose a Directory ”);

list = getFileList (dir);

setBatchMode (true);

for (i = 0; i < list.length; i++) {

path = dir + list [i];

open (path);

run (“Brightness/Contrast.”); setMinAndMax (157, 2669);

run (“Apply LUT”);

run (“Merge Channels.”, “c2 = [“+list [i]+”]”);

run (“Sharpen”);

‘ newdir = getDirectory (“Choose a Directory”);

‘ MkDir newdir

save (path+“-colourised.png”);

setBatchMode (false);

run (“Close”);

}}

#### 2.5.2 Counting code:

macro “Batch Convert to Binary” {

dir = getDirectory (“Choose a Directory”);

list = getFileList (dir);

setBatchMode (true);

for (i = 0; i < list.length; i++) {

path = dir + list [i];

open (path);

run (“8-bit”);

setAutoThreshold ();

run (“Threshold.”);

setThreshold (20, 255);

run (“Convert to Mask”);

setThreshold (255, 255);

run (“Watershed”);

run (“Analyze Particles.”, “size = 400-Infinity circularity = 0.00–1.00 show = Outlines display clear summarize”);

dotIndex = lastIndexOf(path, “.”);

if (dotIndex! = −1)

path = substring (path, 0, dotIndex);//remove extension

save (path+“−20_bin.tif”); close ();

}

}

setBatchMode (false);

### 2.6 Cell treatment

#### 2.6.1 shRNA induction

Lentiviral stable shRNA knockdown cells were seeded 1 day prior to induction with 2 μg/mL of doxycycline (Fisher Scientific). The medium was exchanged every other day, if not stated otherwise. shRNA-mediated knockdown was verified by RT-qPCR.

#### 2.6.2 Cytokine treatment

Lentiviral stable shRNA knockdown cells were seeded 1 day prior to treatment with IFNγ (500 IU/mL for 501-mel and Ma-Mel-61a, 100 IU/mL for Ma-Mel-86c; Imukin, Boehringer Ingelheim) for the specified duration. Control cells were left untreated. Cells were harvested and further analyzed.

### 2.7 Immunostaining and flow cytometry

Cells were harvested at indicated time points post-treatment and washed twice with PBS. For surface staining, cells were incubated at 4°C for 1 h in the dark with anti-HLA-ABC-APC (clone W6/32, Invitrogen) diluted in FACS buffer (10% FBS in PBS). After washing twice with FACS buffer, the cells were fixed with 4% paraformaldehyde. Samples were measured using the SH800S Cell Sorter (Sony Biotechnology). Unstained and untreated cells served as controls. Analysis of the mean fluorescence intensity (MFI) was performed using R. The normalization of the MFI of the treated cells compared to the respective controls gives the fold change as the relative MFI.

### 2.8 Flow cytometry data analysis using R

RStudio (Version 4.3.0) was used for flow cytometry data analysis and the R Script kindly provided by Dr. Tzu-Chen Lin (Technical University of Dortmund). First, Flow Cytometry standard files (FCS) were imported and the Bioconductor packages flowCore (2.0.0) (Ellis, et al., 2017), flowClust (3.26.0) (Lo et al., 2008; Lo et al., 2009), flowDensity (1.22.0) (Malek et al., 2015), flowStats (4.0.0) (Hahne et al., 2017), and ggcyto (1.16.0) (Van et al., 2018) were loaded. The fluorescence intensity data collected from specified populations were subsequently processed using Tidyverse packages (1.3.0). Cell populations were initially identified from t mixture models, followed by singlet cells gating using a robust linear model with rlm. A boundary filter was applied and data was extracted including mean, median and SD values. Data was illustrated as histograms.

### 2.9 RNA-sequencing library preparation

RNA was extracted using TRIzol™ Reagent. Prior to library preparation, rRNA was removed using QIAseq FastSelect -rRNA HMR Kit according to the manufacturer’s guidelines. Libraries were prepared using QIAseq Stranded RNA Lib Kit UDI according to the corresponding handbook. Libraries were submitted to the Sequencing Core Facility at the Max Planck Institute for Molecular Genetics, Berlin, for sequencing on an Illumina-NovaSeq 6000 PE150 yielding at least 3 × 10^7^ reads for each sample.

### 2.10 RNA sequencing analysis

The raw fastq files were processed using the zarp pipeline (Katsantoni et al., 2021), which uses FastQC, zpca and MultiQC (Andrews, 2010; Ewels et al., 2016) (https://github.com/zavolanlab/zpca) for quality control and the adapters are trimmed using Cutadapt (Martin, 2011). The reads were mapped to the human genome (hg38, Genome Reference Consortium GRCh38) using STAR (Dobin et al., 2013) and quantized using Salmon (Patro et al., 2017). The final output is a count matrix which is used as input for the R package DESeq2 (Love et al., 2014) to identify differentially expressed genes. Genes with a logFoldChange>1 and adjusted *p*-value < 0.05 are used for further analysis. The volcano plot was generated using the EnhancedVolcano R-package (Blighe, 2018) and the ComplexHeatmap (Gu et al., 2016) package was used for generating the heatmaps. The gene sets were obtained using the msigdbr (https://CRAN.R-project.org/package=msigdbr) R-package and clusterProfiler (Wu et al., 2021) and enrichplot (Yu, 2018) were used for generating the Gene-Set Enrichment plots. Detailed quality control information involving sequencing depth, mapping rate and coverage of each sample are listed in Supplementary Table S2.

### 2.11 RNA pulldown

GRASLND RNA pulldown was performed according to Dimartino et al. (2018) with certain changes. Human melanoma 501-mel cells were harvested and cell lysates were obtained using lysis buffer (50 mM HEPES, pH 7.5, 150 mM KCl, 0.5% IGEPAL, 0.5 mM DTT, 2 mM EDTA), supplemented with 1 × complete protease inhibitor cocktail (Roche). Cells from a fully confluent 15 cm plate were used for each sample (probe set “odd,” probe set “even” and probe set “lacZ Control”). Dynabeads™ MyOne™ Streptavidin C1 magnetic beads (Thermo Fisher) were prepared according to the manufacturer’s protocol for RNA applications. Beads were blocked (100 μg/mL salmon sperm DNA, 5% BSA, 0.02 μg/mL Heparin in lysis buffer) at 4°C on a rotating wheel for 30 min and were subsequently washed three times with 1 mL hybridization buffer (50 mM Tris-HCl, pH 7.4, 150 mM NaCl, 1 mM MgCl_2_, 0.05% IGEPAL, 10 mM EDTA, 1 mM DTT) supplemented with protease and RNase inhibitor (Promega). The lysates were pre-cleared by incubation with 20 µL of previously blocked beads in hybridization buffer at 4°C while rotating for 30 min. A mix of five biotinylated oligonucleotides per probe set (60 μM each oligonucleotide) was added to the pre-cleared lysates and incubated at room temperature while rotating for 80 min. To each sample, 200 μL of blocked beads were added and incubated at room temperature using a rotating wheel for 30 min. The beads were washed five times with 1 mL hybridization buffer. In the final washing step, beads were divided equally for RNA and protein analysis. The elution from the beads was performed using TRIzol™ Reagent and 1 × NuPAGE LDS sample buffer, respectively, followed by RNA extraction or Western blot analysis. The sequences of the biotinylated oligonucleotides belonging to probe set “odd,” probe set “even” and lacZ control are listed in Supplementary Table S3. The same lacZ sequences were used as described by Dimartino et al. (2018).

### 2.12 Generation of stable cell lines expressing inducible shRNAs

#### 2.12.1 Cloning

Short hairpin RNA (shRNA) sequences targeting GRASLND lncRNA (shGRAS1, shGRAS2) were designed using the InvivoGen siRNA Wizard Software^3.1^. An shRNA targeting the lacZ mRNA (target sequence 5-ctcggcgtttcatctgtgg-3′) was used as a negative control (shCtr) using a sequence from Feng et al. (Feng et al., 2009). Selected shRNAs (target sequence shGRAS1: 5′-gatccaagcacagcaattt-3′, shGRAS2: 5′-cttagagaacaagggttataa-3′) were cloned into lentiviral vector Tet-pLKO-puro (Addgene #21915) based on the instructions of the protocol “The “all-in-one” system for the inducible expression of shRNA” (Wiederschain et al., 2009). In brief, the backbone vector was double digested with AgeI and EcoRI restriction enzymes and gel purified. DNA oligos were annealed and cloned into digested vector using T4 DNA ligase following standard protocol. Cloning products were transformed in chemically competent One Shot^®^ Stbl3™cells (Thermo Fisher) according to standard protocols. Successful cloning was verified by colony PCR and subsequent Sanger Sequencing.

#### 2.12.2 Lentivirus production

4 × 10^6^ Lenti-X 293T cells were seeded per 10 cm culture dish 1 day prior to transfection and incubated overnight. Lenti-X 293T cells were transfected with viral packaging plasmid psPAX2 (7.4 μg), envelope plasmid pMD2.G (5.5 μg) and lentiviral vector Tet-pLKO-puro containing GRASLND knockdown (shGRAS1, shGRAS2) or lacZ-targeting negative control (shCtr) sequences (11.25 μg) using PEI reagent (1 mg/mL). Lentivirus-containing supernatants were collected 48, 72 and 96 h post transfection, centrifuged and filtered through 0.2 μm syringe filters. Viral supernatants were concentrated using Amicon® Ultra-15 Centrifugal Filter Unit (Millipore) to a total volume of 500 µL and stored at −80°C until further use.

#### 2.12.3 Lentivirus transduction

Lentiviral transduction was performed in 501-mel, Ma-Mel-86c and Ma-Mel-61a cells. Cells were seeded in a 6-well plate at 2 × 10^5^ cells/well 1 day prior to transduction and incubated overnight. Medium was exchanged containing 8 μg/mL of polybrene and 50 μL of concentrated lentiviral supernatant (1/10 of total virus) was added drop wise. Medium replacement was performed after 16–18 h. Stable transductant pools of GRASLND knockdown or non-targeting control cells were obtained by selection with puromycin (2 μg/mL) for 3 days, beginning 48 h post-transduction.

### 2.13 TCGA data analysis

RNA-Seq data was obtained from TCGA-SKCM database. Gene expression data of 471 melanoma patients from the TCGA project (produced by STAR concurrent with alignment) was obtained from GDC TCGA-SKCM data portal (https://portal.gdc.cancer.gov/projects/TCGA-SKCM) released in August 2023. The data for healthy patients was sourced from GTeX database (https://gtexportal.org/home/) with a total of 701 healthy samples. The differential gene expression analysis was done with the DESeq2 (Love et al., 2014) package. Genes with Log2FoldChange>1 and multiple testing corrected *p*-value<0.1 (Benjamini–Hochberg) were classified as differentially expressed.

The volcano plots were generated using the EnhancedVolcano (Blighe, 2018) and TPM counts were used for the violin plot. The significance level was tested using the Wilcoxon’s rank-sum test for a total of 471 tumor samples and 701 healthy control samples.

Additionally, the tumor data from TCGA was divided into two subsets based on the expression level of GRASLND (expression>=median or expression < median) in VST transformed (from DESeq2) expression data. The data was utilized to conduct survival analysis employing the Kaplan-Meier method through the TCGA Biolinks package (Colaprico et al., 2016). Human melanoma samples (TCGA dataset, SKCM, n = 471 patients) were divided into immunological hot and cold tumors based on the presence of CD8A transcripts. The volcano plot showed the fold changes and *p* values of transcripts in hot tumors (CD8A, top 10%) *versus* cold tumors (CD8A, bottom 10%), as performed by Li et al. (2021). Statistical analysis was performed using two-sided t-tests.

Correlation of GRASLND with other genes was calculated on normalized counts obtained from the Wouters data set (Wouters et al., 2020) using Spearman correlation.

### 2.14 Statistical analysis

Independent biological replicates were performed for cell-based assays and data are shown as mean ± SD, unless stated otherwise. For RT-qPCR, three technical replicates per sample were used and data are represented as mean ± SD. Statistical tests were performed using GraphPad Prism software version 9 and the integrated development environment RStudio. The two-tailed unpaired *t*-test was applied for comparing experimental groups. A value of *p* < 0.05 was considered statistically significant.

## 3 Results

### 3.1 GRASLND is predominantly expressed in differentiated melanoma cells

**FIGURE 1:**
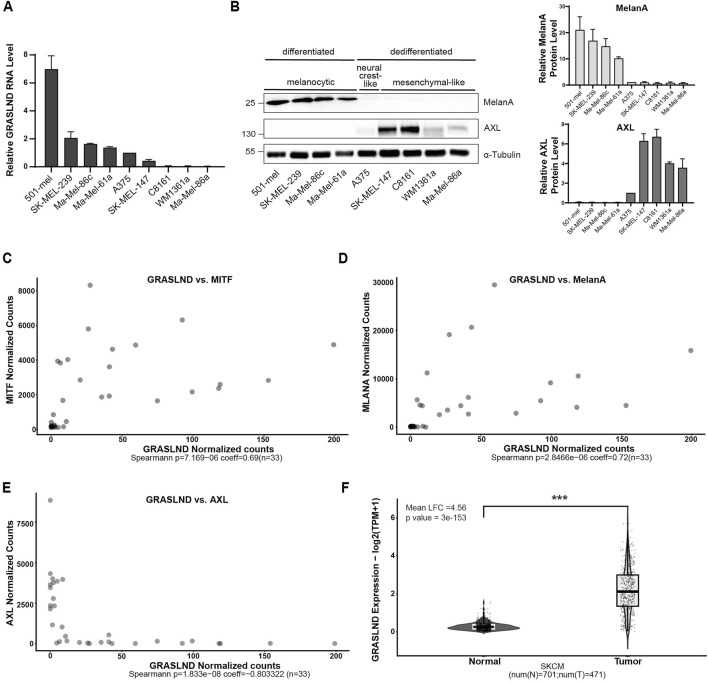
GRASLND is predominantly expressed in differentiated melanoma cells. **(A)** Relative GRASLND RNA expression of nine melanoma cell lines. RNA levels were determined using RT-qPCR and normalized to GAPDH mRNA levels. Expression levels of each replicate was normalized to neural crest-like cell line A375. Significance tests for all cell lines are listed in Supplementary Table S4 n = 3 independent biological replicates. **(B)** Protein levels of MelanA and AXL of nine melanoma cell lines determined by Western blotting and grouping into different melanoma cell states. α-Tubulin, loading control (left). Quantification of MelanA and AXL protein levels. Protein levels of each replicate was normalized to the expression level in cell line A375 (right). Significance tests for all cell lines are listed in Supplementary Tables S5 and S6. Representative blot out of n = 3 independent biological replicates. **(C–E)** Transcript correlation analysis of 33 melanoma samples from a data set (Wouters et al., 2020) between GRASLND and MITF **(C)**, MelanA **(D)** and AXL **(E)** gene expression. Spearman coefficient = 0.69 (MITF), 0.72 (MelanA) and −0.80 (AXL). *p*-value = 7.2 × 10^−6^ (MITF), *p* = 2.8 × 10^−6^ (MelanA) and *p* = 1.8 × 10^−8^ (AXL). **(F)** GRASLND is significantly overexpressed in skin tumors (TCGA, SKMC, n = 471) compared to normal skin tissues (GTEx database, n = 701). *p*-value = 3 × 10^−153^.

Given its identification as a key regulator in mesenchymal stem cells (MSCs), where it suppresses IFNγ signaling (Huynh et al., 2020) and considering its emerging role in cancer biology (Wu et al., 2024), we investigated the function of lncRNA GRASLND in the context of melanoma. Recognizing the role of melanoma cell differentiation status in therapy resistance (Mehta et al., 2018; Lee et al., 2020) and tumor aggressiveness (Hoek et al., 2006; Hoek et al., 2008), we first aimed to examine whether GRASLND expression varies depending on cell state. Therefore, we determined the expression of GRASLND lncRNA across nine melanoma cell lines (Figure 1A; Supplementary Table S4). These melanoma cell lines were tested for their cell states by measuring the protein expression of melanocytic marker MelanA, alongside the dedifferentiation marker AXL, a receptor tyrosine kinase (RTK) associated with the mesenchymal-like and drug-resistant melanoma cell state (Figure 1B) (Müller et al., 2014; Rambow et al., 2019). Interestingly, all differentiated melanoma cell lines, such as 501-mel, SK-MEL-239, Ma-Mel-86c and Ma-Mel-61a, expressed significantly higher GRASLND levels in contrast to all dedifferentiated, mesenchymal-like and AXL^high^ cell lines SK-MEL-147, C8161, WM1361a and Ma-Mel-86a (Supplementary Tables S4–S6). Based on this observation, we performed a correlation analysis of GRASLND with the lineage-specific transcription factor MITF, its target MelanA and the dedifferentiation marker AXL using bulk RNA sequencing data (Wouters et al., 2020). This data set comprises 33 melanoma cultures, grouped and investigated for melanocytic, intermediate, neural-crest-like and mesenchymal-like cell states. Spearman correlation analysis revealed a positive correlation of GRASLND with melanocytic markers MITF and MelanA with Spearman coefficients of 0.69 (*p* = 7.2 × 10^−6^) and 0.72 (*p* = 2.8 × 10^−6^), respectively (Figures 1C,D). In line, GRASLND is inversely correlated with RTK AXL, having a Spearman coefficient of −0.80 (*p* = 1.8 × 10^−8^) (Figure 1E), supporting our findings of a correlation between GRASLND expression and the melanoma cell state. In addition, the expression level of GRASLND in skin cutaneous melanoma (SKCM-TCGA, n = 471) is significantly higher (mean Log2FC = 4.56, p = 3 × 10^−153^) than in normal skin tissues (GTEx database, n = 701), indicating a pathological relevance in this malignancy (Figure 1F).

### 3.2 GRASLND knockdown impairs melanoma cell proliferation

**FIGURE 2:**
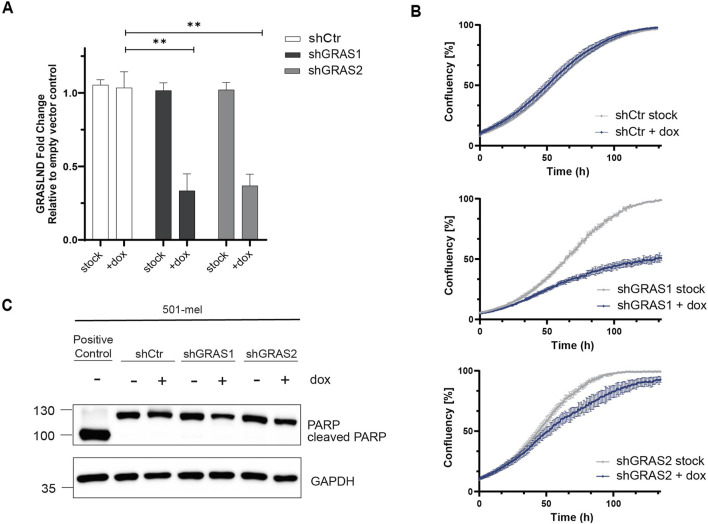
GRASLND knockdown impairs melanoma cell proliferation. **(A)** Relative GRASLND expression in 501-mel shRNA knockdown cells compared to empty vector control cells after induction with doxycycline (2 μg/mL) for 72 h, determined by RT-qPCR and normalized to GAPDH mRNA levels. Fold change of GRASLND levels of shRNA-knockdown cells relative to the empty vector control cells are given as mean ± SD. *p*-values by two-sided *t*-test. n = 3 independent biological replicates. **(B)** Impact of GRASLND knockdown on cell growth. 501-mel cells were either treated with doxycycline or left untreated and cell confluency was monitored using IncuCyte S3. One representative growth curve of 3 independent biological replicates per knockdown cell line is shown. **(C)** PARP cleavage assay. GRASLND knockdown cells were induced for 7 days with doxycycline (2 μg/mL) followed by Western blot analysis using an anti-PARP antibody. Cells treated with the apoptosis-inducing reagent etoposide (150 μM, Sigma Aldrich) served as positive control. GAPDH, loading control. Representative blot out of n = 3 independent biological replicates.

To investigate the role of lncRNA GRASLND in melanoma, we generated doxycycline-inducible knockdown cell lines using two small hairpin RNAs (shGRAS1 and shGRAS2) and a non-targeting control shRNA (shCtr). The human melanoma cell line 501-mel was selected as a differentiated, melanocytic cell model exhibiting the significantly highest GRASLND expression among all tested cell lines (Figures 1A,B; Supplementary Table S4). Both doxycycline-induced shRNAs, shGRAS1 and shGRAS2, reduced GRASLND expression significantly by 71% and 67%, respectively, compared to control shRNA (Figure 2A). Interestingly, live cell imaging revealed that GRASLND knockdown impaired cell proliferation (Figure 2B). To determine whether GRASLND knockdown affects cell survival, a PARP1 (Poly (ADP-ribose) polymerase 1) cleavage assay was performed indicating apoptosis. Cells were treated with doxycycline to induce shRNA expression for a duration of 7 days or with apoptosis inducer etoposide as a positive control. GRASLND knockdown did not induce PARP1 cleavage in 501-mel cells (Figure 2C). Also live cell imaging did not indicate enhanced cell death upon GRASLND knockdown. Overall, these findings demonstrated that GRASLND knockdown induced a transition to a non-proliferative melanoma cell state without inducing cell death.

### 3.3 GRASLND knockdown induces melanoma phenotype switching

**FIGURE 3:**
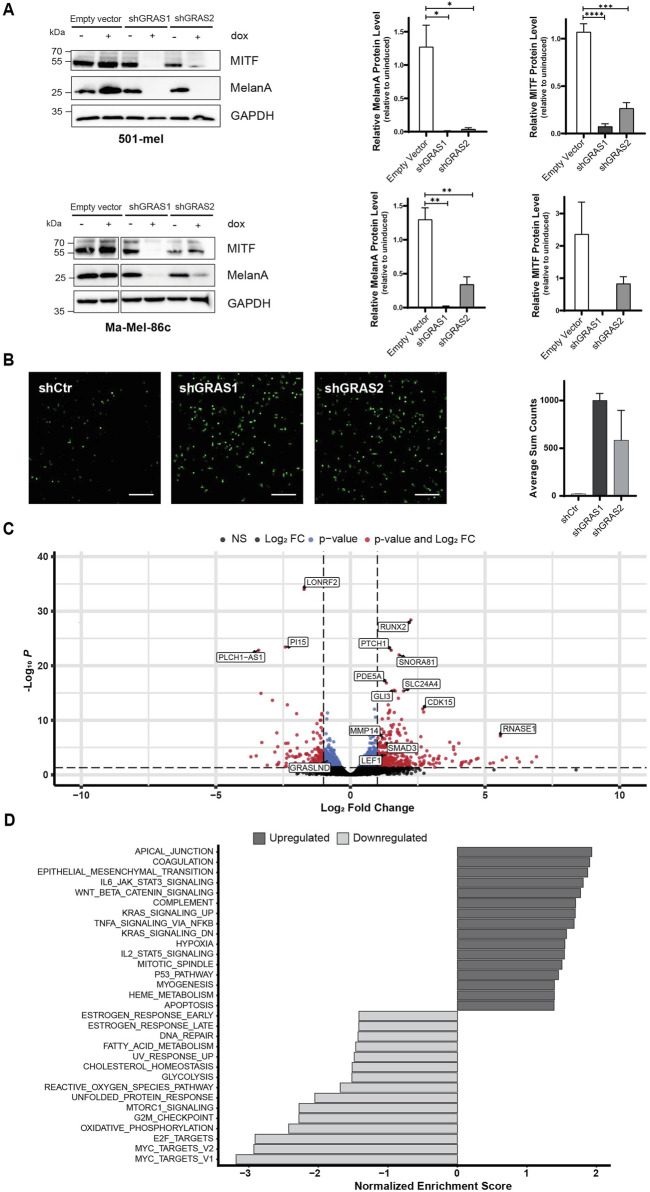
GRASLND knockdown induces melanoma phenotype switching. **(A)** Expression of melanoma differentiation marker MITF and MelanA after GRASLND knockdown in 501-mel (top) and Ma-Mel-86c cells (bottom) induced with doxycycline (2 μg/mL) for 72 h analyzed by Western blotting (left), GAPDH as loading control. Quantification of MITF and MelanA levels given as mean ± SEM. *p*-values by two-sided *t*-test (right). Representative blot out of n = 3 independent biological replicates. **(B)** Representative images (left) and quantification (right) of 501-mel shRNA-knockdown cell lines induced with doxycycline (2 μg/mL) for 72 h and subsequently subjected to a Transwell invasion assay. n = 2 independent biological replicates. Scale bar = 25 µm. **(C)** Volcano plot of differentially expressed genes after shRNA-mediated GRASLND knockdown by shGRAS1 and shGRAS2 compared to shCtr using RNA-Sequencing. Stable shRNA knockdown cells of cell line 501-mel were induced for shRNA expression for 72 h. Medium was exchanged every day. The two vertical lines represent the thresholds for a log2FoldChange, while the horizontal line denotes the threshold for statistical significance (adjusted *p* < 0.05). Red dots indicate genes with statistically significant up- or downregulation. **(D)** Gene set enrichment analysis (GSEA) of affected pathways after GRASLND knockdown using the Hallmark pathway gene sets (https://CRAN.R-project.org/package=msigdbr). Top 16 enriched pathways of upregulated and top 15 enriched pathways of downregulated genes are shown. NS = non-significant, FC = Fold Change.

Phenotypic melanoma cell states are defined by specific gene expression profiles, as firstly described by Hoek et al. (2008). Based on this, the slow-proliferative melanoma cell state is characterized by a low level of the lineage-specific transcription factor MITF. Therefore, the expression levels of MITF and its target MelanA in response to GRASLND downregulation were investigated. Indeed, upon GRASLND knockdown, in 501-mel (Figure 3A, top) and Ma-Mel-86c (Figure 3A, bottom) cells, the expression of MelanA was significantly decreased. For MITF, a significant reduction in protein levels was observed in 501-mel cells. The MITF^low^-phenotype is described as slow growing and highly invasive (Hoek et al., 2006). As invasiveness plays a pivotal role in metastatic spreading and melanoma progression, we investigated whether the observed phenotype displayed invasive properties in an *in vitro* Transwell invasion assay. Both shRNAs increased the invasion capacity of 501-mel cells by a fold change of 48.8 ± SD = 2.5 and 28.5 ± SD = 10.8, respectively, compared to the control shRNA (Figure 3B). Taken together, these observations suggest the involvement of GRASLND in controlling melanoma plasticity. GRASLND knockdown induced a phenotypic switch from a proliferative, less invasive and differentiated melanoma cell state towards a non-proliferative, highly invasive and dedifferentiated state.

To determine the impact of GRASLND on the transcriptome of 501-mel cells, RNA sequencing was performed 3 days after doxycycline-induced shRNA knockdown. Principal component analysis (PCA) revealed distinct clustering patterns of control samples and shRNA-knockdown samples, as expected, explaining 64% variance on PC1 (Supplementary Figure S1A). Transcriptomic analysis upon knockdown revealed the total number of 549 differentially expressed genes (Fold change>1/<−1, adjusted *p*-value < 0.05). Of those genes, 393 were upregulated, whereas 156 genes were downregulated (Figure 3C). The differentially expressed genes were further analyzed by gene set enrichment analysis (GSEA) using the HALLMARK pathway gene sets (Figure 3D). Among the top 15 downregulated gene sets we found a number of pathways involved in cell cycle regulation, such as MYC targets, E2F targets and G2M checkpoint. Genes within these gene sets include CDK1, CDK4 and CDKN3, supporting our previous findings of impaired proliferation upon GRASLND knockdown as it suggests a global inhibition of cell cycle progression (Supplementary Table S7; Figure 2B). In line with our findings on GRASLND knockdown-mediated phenotype switching, one of the most upregulated HALLMARK gene set was the epithelial to mesenchymal transition (EMT). Further gene signatures described to induce dedifferentiation in melanoma, such as JAK-STAT3- (Swoboda et al., 2021), TNFα- (Rossi et al., 2018) and WNT signaling (Eichhoff et al., 2011), were significantly enriched in the present data set. Additional analysis revealed the upregulation of key components of the mentioned pathways, such as STAT3, NFKB2 and LEF1 (Supplementary Table S8; Figure 3C). Remarkably, upregulation of STAT3 expression upon GRASLND knockdown was confirmed on protein level (Supplementary Figure S1B), suggesting a potential STAT3-mediated downregulation of MITF expression (Swoboda et al., 2021). In sum, the transcriptomic data confirm a role for GRASLND in melanoma differentiation, as its downregulation induces switching towards a dedifferentiated, highly invasive cell state and points towards potentially critically pathways involved.

### 3.4 GRASLAND expression is enriched in immune cold melanoma

**FIGURE 4:**
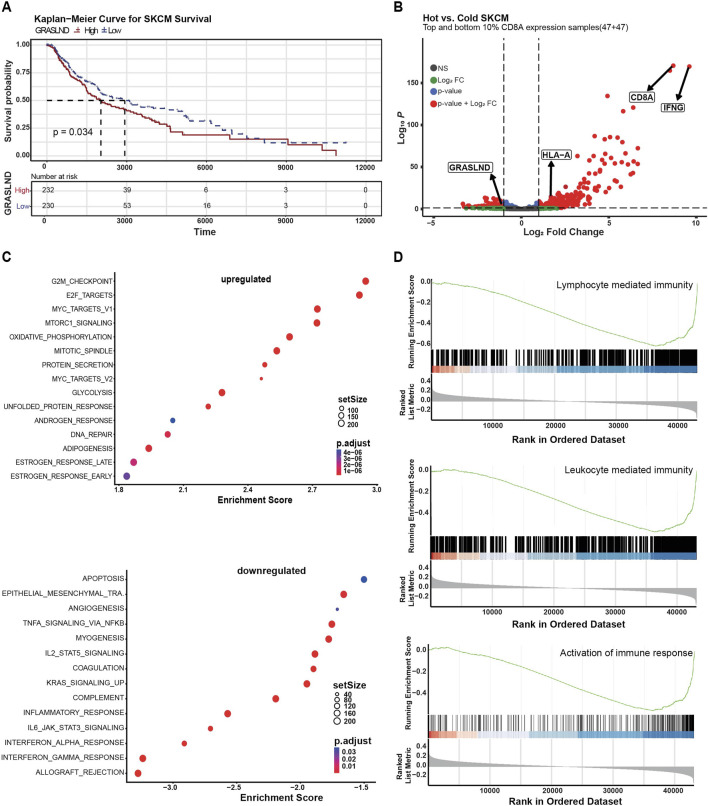
GRASLND is enriched in immune cold melanoma. **(A)** Melanoma patient survival plot (TCGA, SKMC) based on GRASLND high (n = 232) and low (n = 230) groups. Two-sided log-rank test for *p*-value. **(B)** Human melanoma samples (TCGA, SKCM) were divided into hot and cold tumor groups based on CD8A transcript levels, indicating the abundance of immune infiltrates. Volcano plot shows top and bottom 10% CD8A expression samples and fold changes and *p* values of transcripts. Statistical analysis was performed using two-sided t-tests. **(C)** Gene set enrichment analysis of GRASLND^high^ melanoma patient samples (n = 232) tumors using the Hallmark pathway gene sets. Top 15 upregulated (top) and top 14 downregulated (bottom) pathways are shown. **(D)** Single gene set enrichment of GRASLND^high^ melanoma patient samples (n = 232) for genes involved in the activation of immune response, leucocyte-mediated immunity and lymphocyte mediated immunity. The curves represent the running sum of the enrichment scores and the bars represent the position of genes associated with specific pathways. In the bottom part, the distribution of fold change along with the gene list is shown.

Since we found that GRASLND downregulation induces melanoma phenotype switching, which is associated with melanoma progression (Alonso et al., 2007; Li et al., 2015) and therapy resistance (Konieczkowski et al., 2014; Müller et al., 2014; Mehta et al., 2018; Lee et al., 2020), we were interested in its potential clinical relevance. To validate this, we analyzed human melanoma patient data from the TCGA database grouped by their tumoral GRASLND expression with regard to the overall survival. Tumors showing high GRASLND expression levels were significantly associated with impaired patient survival (p = 0.034) (Figure 4A). Moreover, we grouped human melanoma samples from the TCGA database (SKCM) into immunological “hot” and “cold” tumor groups based on CD8A transcript levels serving as an indicator for tumor infiltrating lymphocytes (Gajewski et al., 2017). In fact, GRASLND expression was enriched in cold tumors (Figure 4B).

GSEA of GRASLND^high^ tumors (n = 232 patient samples) using the HALLMARK pathway gene sets revealed IFNα-, IFNγ- and inflammatory, as well as IL2-STAT5-and IL6-STAT3-signaling responses among the top nine downregulated pathways in a large patient cohort (Figure 4C, bottom), the latter in line with our *in vitro* observations in GRASLND knockdown experiments (Figure 3D). Furthermore, we found a negative correlation of GRASLND expression and gene signatures of pro-inflammatory cellular processes (Figure 4D). Thus, we concluded that GRASLND is an immune-related lncRNA that is of clinical relevance and may contribute to tumor immune evasion.

### 3.5 GRASLND impairs HLA-I upregulation under IFNγ

**FIGURE 5:**
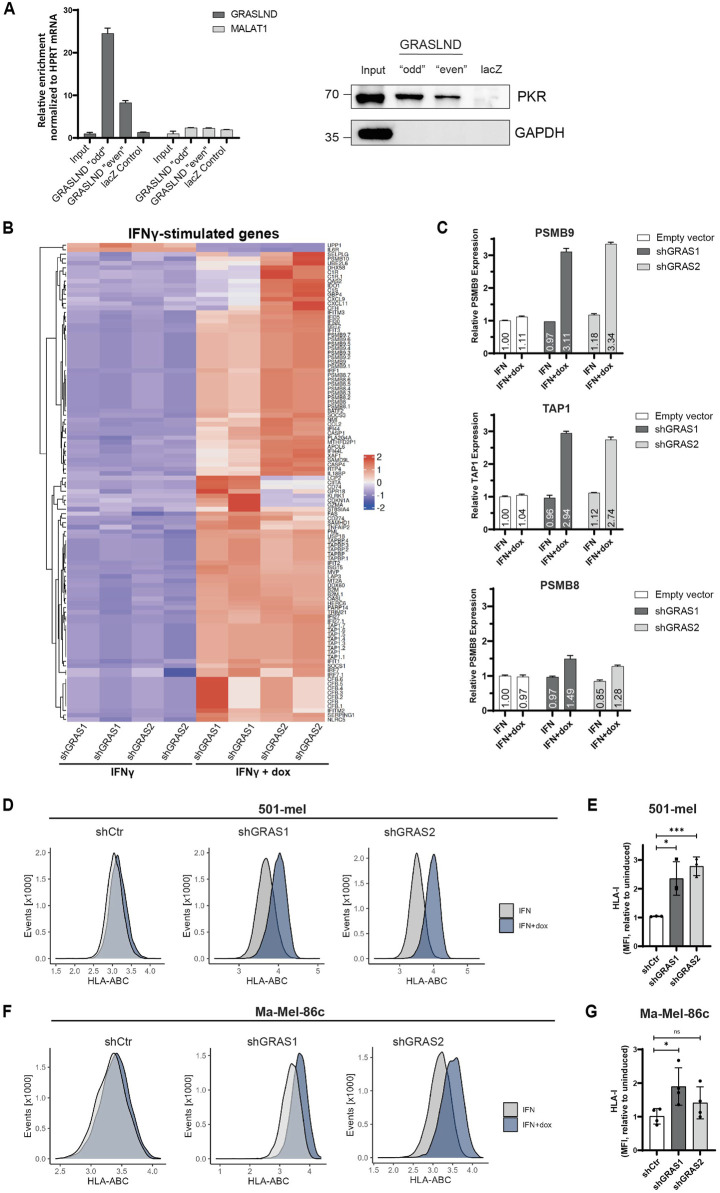
GRASLND impairs HLA-I upregulation under IFNγ. **(A)** RNA pulldown of GRASLND followed by Western blot analysis. Left panel shows GRASLND enrichment validated by RT-qPCR and normalized to HPRT1 mRNA levels. Right panel shows Western blot of PKR subsequent to GRASLND pulldown. Representative data of three independent experiments. **(B)** Heatmap of differentially expressed IFNγ-stimulated genes after IFNγ treatment and shRNA-mediated GRASLND knockdown for 6 days using RNA-Sequencing. **(C)** Relative RNA expression confirmation of ISGs examples PSMB9, TAP1 and PSMB8 after GRASLND knockdown and simultaneous treatment with IFNγ for 3 days, determined by RT-qPCR and normalized to GAPDH mRNA levels. **(D)** HLA-I surface expression in 501-mel cells determined by flow cytometry. Representative histograms of control and GRASLND knockdown cell lines from independent biological replicates (n = 3). **(E)** Fold change of MFI of IFNγ-only to IFNγ+dox treated cells given as mean ± SD in 501-mel cells. **(F)** HLA-I surface expression in Ma-Mel-86c cells determined by flow cytometry. Representative histograms of control and GRASLND knockdown cell lines from independent biological replicates (n = 4). **(G)** Fold change of MFI of IFNγ-only to IFNγ+dox treated cells given as mean ± SD in Ma-Mel-86c cells.

Previous studies by Huynh et al. (2020) in mesenchymal stem cells (MSCs) showed an interaction of GRASLND with the interferon-induced, double-stranded RNA-activated protein kinase R (PKR) and hypothesized GRASLND-dependent inhibition of the IFNγ signaling by preventing the STAT-DNA interaction required for gene expression. To validate the direct interaction of GRASLND and PKR, an RNA pulldown assay with subsequent verification of bound PKR protein was performed. For this, two sets (“odd” and “even”) with five biotinylated antisense DNA oligonucleotides, were used to pull-down lncRNA GRASLND using cytoplasmic extracts from 501-mel cells (Supplementary Figure S2A). A probe set of five biotinylated antisense DNA oligonucleotides targeting the lacZ mRNA served as negative control. RT-qPCR analysis proved a 24.5 to 8.3-fold specific GRASLND enrichment with both probe sets ‘odd’ and “even” and no enrichment of the control lncRNA MALAT1 (Figure 5A, left, Supplementary Figure S2B). Importantly, the direct interaction of PKR protein and GRASLND lncRNA in both RNA pull down samples was confirmed, while lacZ control pull-down protein signal remained in background level (Figure 5A, right). Interestingly, PKR protein levels are downregulated upon GRASLND knockdown (Supplementary Figures S2C, D). Applying the hypothesis of IFNγ signaling inhibition by GRASLND to the melanoma background, its knockdown would lead to an elevation of the expression of IFNγ-stimulated genes (ISGs) in response to cytokine treatment. Indeed, treatment of shGRAS1/shGRAS2 501-mel cells with either IFNγ or IFNγ plus doxycycline for 6 days, followed by RNA-Seq analysis, demonstrated a significant upregulation (*p* < 0.05) of a vast number of ISGs after GRASLND knockdown and not the control shRNA (Figure 5B, Supplementary Figure S2E). Among those ISGs we found genes involved in antigen processing and presenting machinery (e.g., TAPBP, TAP1), immunoproteasome genes (PSMB8 and PSMB9), immune cell recruitment (CXCL9, CXCL11) or CD8^+^ T lymphocyte recognition and inhibition (B2M and CD274/PD-L1, respectively). Notably, we confirmed these results for PSMB8, PSMB9 and TAP by RT-qPCR on cells exposed to IFNγ or IFNγ plus doxycycline over 3 days. Indeed, PSMB9 and TAP1 mRNA expression increased upon GRASLND knockdown in IFNγ-treated cells by approximately 3.2- and 2.8-fold, respectively. PSMB8 expression was only slightly enhanced by about 1.5-fold (Figure 5C). Since we found enhanced expression of genes involved in antigen processing and presentation, we analyzed the surface expression of HLA class I molecules on three different melanoma cell lines by flow cytometry. Notably, knockdown of GRASLND in IFNγ-treated 501-mel cells led to a significant increase in HLA-I surface expression by 2- and 3-fold using both shRNAs, respectively (Figures 5D, E). Similarly, upregulation of HLA-I was observed in Ma-Mel-86c cells by 1.5- and 2.0-fold (Figures 5F, G) as well as in cell line Ma-Mel-61a (Supplementary Figures S2F, G), though to a lesser extent. In sum, our findings support the hypothesis that GRASLND inhibits IFNγ signaling in melanoma, suggesting an immune evasion mechanism of melanoma cells by upregulating lncRNA GRASLND.

## 4 Discussion

In recent years, an expanding number of studies have focused on lncRNAs and their role in melanomagenesis, revealing their significance as tumor suppressors, oncogenes or prognostic factors in a cell type- and tissue-specific manner (Melixetian et al., 2022). In this study, we investigated lncRNA GRASLND in the melanoma context. Thus far, GRASLND was reported as a prognostic factor in bladder cancer (Wang et al., 2020; Zhong et al., 2022), gastric cancer (Li et al., 2021), glioblastoma (Liu et al., 2021), head and neck squamous cell carcinoma (Hu et al., 2020), osteosarcoma (Rothzerg et al., 2021), and papillary renal cell carcinoma (Chen et al., 2021) by using bioinformatic pipelines and clinical data from the TCGA database. Functional assays of GRASLND in glioma, gastric and bladder cancer, demonstrated an enhancing effect of GRASLND on proliferation, migration and invasion (Wang et al., 2020; Tong et al., 2021; Li et al., 2021). A newly published study verified that these tumor-promoting functions are also present in melanoma (Yang et al., 2024). In line with those studies, we also found GRASLND upregulation in tumorous compared to healthy samples and an association with poor patient survival. Also congruent with these earlier reports in various cancer types, including melanoma, we noted that GRASLND facilitated cell proliferation. However, in contrast to recent findings that showed GRASLND enhances the invasion ability in certain intermediate state melanoma cell lines (Yang et al., 2024), our research indicates it suppresses the invasive potential of differentiated melanoma cells. We demonstrated, that GRASLND silencing increased invasiveness, reduced proliferation and downregulated melanocytic markers MITF and MelanA, indicating an EMT-like phenotypic switch. We conclude, that the discrepancy of the observed phenotypes after GRASLND knockdown regarding melanoma cell invasiveness can be attributed to their different cell states. The cell lines used by Yang et al., A375 and SK-MEL-28, can be categorized based on their gene expression profiles into the neural-crest-like and intermediate state, respectively, as shown by Wouters et al. (2020). Here, we particularly focused on melanocytic, MITF^high^ cell lines due to its high GRASLND levels, which undergo phenotypic switching towards a dedifferentiated, MITF^low^ cell state upon knockdown. A loss of MITF is accompanied by higher invasiveness, as described in the literature (Hoek et al., 2006; Hoek et al., 2008; Simmons et al., 2017). Consequently, the results of both studies are not mutually exclusive, but instead emphasize the importance of the cellular context and multifunctionality as a typical feature of many lncRNAs (Kung et al., 2013).

The exact mechanism by which downregulation of GRASLND promotes melanoma phenotypic switch demands deeper clarification. However, transcriptomic analysis upon GRASLND knockdown revealed potential signaling pathways that might be involved. GSEA uncovered pathways known to induce phenotypic switch in melanoma, such as IL6-STAT3- (Swoboda et al., 2021), TNFα- (Rossi et al., 2018) and WNT-signaling (Eichhoff et al., 2011). Both, STAT3 and TNFα signaling have been described to drive the transition of differentiated melanoma cells toward dedifferentiation. STAT3 can repress MITF transcription via the transcriptional regulator CEBP (Swoboda et al., 2021). Moreover, STAT3 has been demonstrated to promote metastasis of melanoma cells (Swoboda et al., 2021; Suwei et al., 2022), thereby linking the loss in differentiation with the induction of markers related to the EMT-like cell state transition. An upregulation of STAT3 RNA and protein levels was observed upon GRASLND knockdown (Supplementary Figure S1B; Supplementary Table S8). STAT3 antagonizes MITF and drives melanoma metastasis *in vivo* (Swoboda et al., 2021) suggesting a potential STAT3-mediated dedifferentiation in response to GRASLND downregulation. Additionally, we found increased TGFB1 and SMAD3 RNA levels, key components in the TGF-β1/SMAD signaling (Wrana, 2013) known to promote EMT and metastasis in cancer (Hao et al., 2019; Tuncer et al., 2019). In gastric cancer, GRASLND is induced by TGF-β1 (Li et al., 2021). Overall, this suggests that phenotypic switch mechanisms may involve a complex interplay of several pathways based on the multifunctionality of lncRNAs that can also apply for GRASLND.

The discovery of GRASLND’s upregulation in melanoma and its impact on phenotypic switching, along with affected pathways, point towards potential clinical relevance. Indeed, GRASLND overexpression correlates with poor clinical prognosis. Unlike the findings in other cancer types, the increased mortality and the negative impact on survival cannot be attributed to a promoting effect on cancer cell migration and invasion. We showed that GRASLND expression is associated with the differentiated, proliferative and non-invasive phenotype in melanoma. Thus, the poorer prognosis cannot be explained by the ability to trigger increased metastasis. Nevertheless, we found a negative association of GRASLND expression and tumor immunosurveillance. Clinical data analysis from melanoma patients unveiled enrichment of GRASLND in immunologically ‘cold tumors’ characterized by reduced immune infiltrates and lower response to immune checkpoint inhibitors (ICIs) (Gajewski et al., 2017). GSEA of GRASLND^high^ tumors demonstrated a negative correlation with gene signatures of immune responses. This indicates that GRASLND’s negative impact on patient survival is probably a result of its adverse effects on tumor immunogenicity and its role as a potential immune evasion mechanism in melanoma patients.

Functional studies in MSCs discovered an interaction of GRASLND with PKR, thereby suppressing IFNγ signaling (Huynh et al., 2020). Based on this and our RNA-Seq results on the IFNγ-associated gene sets, we suspected a suppressor role of GRASLND on IFNγ signaling in melanoma cells. We demonstrated a direct physical interaction of GRASLND with PKR along with inhibitory effects on the IFNγ signaling and HLA-I cell surface expression. PKR is described to form a complex with STAT1, modulating its transcriptional activity by inhibiting STAT1-DNA binding (Wong et al., 1997; Huynh et al., 2020). Typically, the activation of STAT1 by IFNγ results in the dissociation of the PKR/STAT1 complex (Wong et al., 1997; Wong et al., 2001). Drawing from our findings and published data in MSCs, we hypothesize that GRASLND serves to uphold the PKR/STAT1 complex despite active IFNγ signaling. Consequently, reduction of GRASLND leads to the disassembly of the complex, allowing STAT1 homodimers to bind to the DNA and initiate the transcription of ISGs.

The findings of increased HLA-I upregulation under IFNγ suggest enhanced immunogenicity upon GRASLND knockdown. One of the most extensively studied cell-intrinsic immune escape mechanisms of tumors is the reduction of HLA expression required for melanoma recognition by cytotoxic CD8^+^ T lymphocytes (CTLs). HLA-I antigen presentation is upregulated by IFNγ signaling, however, immunotherapy resistance can also develop due to defects in this cytokine pathway, resulting in the evasion from CTL surveillance (Kaplan et al., 1998; Zaretsky et al., 2016; Lawson et al., 2020). Although many resistance mechanisms to immunotherapy have been identified, investigation of novel immune escape mechanisms hold significant clinical relevance. In the past years, increasing evidence of a direct or indirect regulation and modulation of immune checkpoint molecules by lncRNAs has been provided, demonstrating the impact of lncRNAs on the efficacy of ICI response in melanoma patients. For instance, LINC00473 has been reported as a regulator of PD-L1 expression via a miRNA sponge mechanism (Zhou et al., 2019; Vishnubalaji et al., 2020). In this study, lncRNA GRASLND seems to have a direct impact on melanoma antigen presentation by inhibiting IFNγ signaling and thus impairs the enhancement of the antigen presentation by IFNγ. We propose that melanoma cells upregulate GRASLND to inhibit this pathway and thus evade immune cell recognition. These observations give reasons for the high prevalence of GRASLND expression in immunologically “cold tumors.” The observed immunological functions of GRASLND in melanoma are supported by a bioinformatical prediction study in gastric cancer, that identified GRASLND as an immune-related lncRNA with a negative prognostic factor for ICI response (Ding et al., 2021).

The contemplation of GRASLND as a therapeutic target aims to potentially enhance immunogenicity by increasing HLA-I antigen processing and presenting machinery (HLA-I-APM) in response to IFNγ, but also increase the multifaceted anti-tumor effects of IFNγ signaling, such as inducing apoptosis and inhibiting angiogenesis (Jorgovanovic et al., 2020). However, its impact on dedifferentiation must be considered, which may mitigate the beneficial effects of increased immunity. Generally, melanoma cell dedifferentiation is linked with reduced immunogenicity towards CTLs and resistance to immunotherapy (Mehta et al., 2018; Lee et al., 2020). Nevertheless, it is still not fully understood whether the immune evasive properties or the capacity to preserve a differentiated, proliferative phenotype of GRASLND is decisive for therapy efficacy. The specific underlying mechanisms need further investigations, particularly with regard to its interaction with PKR and its effects on STAT1-mediated transcription of ISGs.

## 5 Conclusion

**FIGURE 6:**
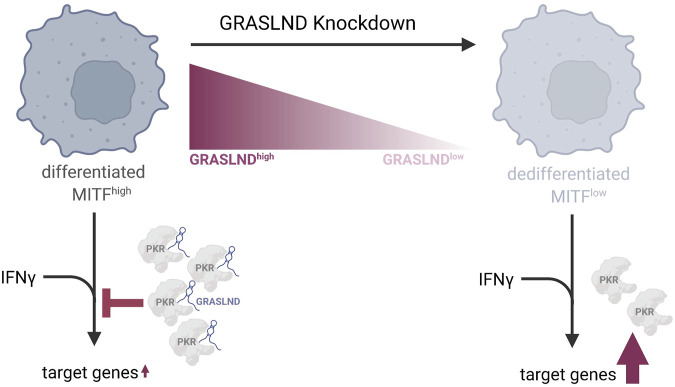
Model of GRASLND’s function in melanoma differentiation and immune response. GRASLND is predominantly expressed in differentiated, melanocytic and MITF^high^ melanomas. A knockdown of this lncRNA induces phenotype switching towards a dedifferentiated cell state, characterized by low MITF levels and reduced PKR expression. Further, elevated GRASLND levels appear to impair melanoma antigen presentation by attenuating the response to IFNγ through direct interaction with PKR, which suppresses STAT1-mediated transcription. Downregulation of GRASLND leads to the enhancement of IFNγ target genes expression, indicating that melanoma cells may upregulate this lncRNA as a mechanism to evade immune cell recognition. Figure created with Biorender.com.

In conclusion, our work on lncRNA GRASLND in melanoma revealed its function as a barrier to the invasive EMT-like phenotypic switch and an association with a differentiated and proliferative melanoma cell state. Despite lncRNA’s tissue- and cell type-specificity, we found an immune-relevant role of GRASLND in suppressing the IFNγ signaling in melanoma, and thus influencing the HLA-I-APM (Figure 6). This elucidates GRASLND’s involvement in immune evasion and its contribution to the poor clinical prognosis. From our study, we conclude that GRASLND could serve as a valuable prognostic biomarker due to its immune-related function and impact on patient survival.

## Data Availability

The RNA sequencing (RNA-seq) data generated and analyzed during the current study are available in the Gene Expression Omnibus (GEO) repository, under accession number GSE273022. Patient data mining was performed using the publicly available RNA-seq data from TCGA-SKCM database. Gene expression data of 471 melanoma patients from the TCGA project was obtained from GDC TCGA-SKCM data portal (https://portal.gdc.cancer.gov/projects/TCGA-SKCM) released in August 2023. The data for healthy patients was sourced from GTeX database (https://gtexportal.org/home/) with a total of 701 healthy samples.
