# Supplementary material for: Long non-coding RNA GRASLND links melanoma differentiation and interferon-gamma response
Source: Front Mol Biosci. 2024 Sep 27;11:1471100. doi: 10.3389/fmolb.2024.1471100 (PMC11466874; doi:10.3389/fmolb.2024.1471100)
Supplement: Supplementary file 1 [file Table5.pdf]

Table S5: *Significance testing of MelanA protein expression was performed across nine melanoma cell lines (see Figure 1B, top). The mean differences in MelanA protein expression measured by western blotting, were calculated from three biological replicates for each cell line, normalized to melanoma cell line A375 and compared against all other cell lines using a two-sided unpaired t-test. p-values marked in red indicate statistical significance, with a threshold of p<0.05.*

Cell Line 1	Cell Line 2	p-value	Mean Cell Line 1	Mean Cell Line 2	t-Statistic
501-mel	SK-MEL-239	0.57002	20.987	16.838	0.618
501-mel	Ma-Mel-86c	0.34574	20.987	14.699	1.068
501-mel	Ma-Mel-61a	0.10012	20.987	10.118	2.131
501-mel	SK-MEL-147	0.01677	20.987	0.939	3.953
501-mel	C8161	0.01591	20.987	0.616	4.017
501-mel	WM1361a	0.01642	20.987	0.779	3.979
501-mel	Ma-Mel-86a	0.01598	20.987	0.669	4.011
SK-MEL-239	Ma-Mel-86c	0.70936	16.838	14.699	0.400
SK-MEL-239	Ma-Mel-61a	0.20639	16.838	10.118	1.507
SK-MEL-239	SK-MEL-147	0.02293	16.838	0.939	3.592
SK-MEL-239	C8161	0.02150	16.838	0.616	3.664
SK-MEL-239	WM1361a	0.02235	16.838	0.779	3.621
SK-MEL-239	Ma-Mel-86a	0.02161	16.838	0.669	3.659
Ma-Mel-86c	Ma-Mel-61a	0.21138	14.699	10.118	1.486
Ma-Mel-86c	SK-MEL-147	0.01052	14.699	0.939	4.537
Ma-Mel-86c	C8161	0.00972	14.699	0.616	4.642
Ma-Mel-86c	WM1361a	0.01026	14.699	0.779	4.570
Ma-Mel-86c	Ma-Mel-86a	0.00972	14.699	0.669	4.642
Ma-Mel-61a	SK-MEL-147	0.00024	10.118	0.939	12.414
Ma-Mel-61a	C8161	0.00021	10.118	0.616	12.801
Ma-Mel-61a	WM1361a	0.00029	10.118	0.779	11.819
Ma-Mel-61a	Ma-Mel-86a	0.00017	10.118	0.669	13.600
SK-MEL-147	C8161	0.55530	0.939	0.616	0.643
SK-MEL-147	WM1361a	0.79350	0.939	0.779	0.280
SK-MEL-147	Ma-Mel-86a	0.56358	0.939	0.669	0.629
C8161	WM1361a	0.79029	0.616	0.779	-0.284
C8161	Ma-Mel-86a	0.90848	0.616	0.669	-0.122
WM1361a	Ma-Mel-86a	0.84002	0.779	0.669	0.215
