# Supplementary material for: Long non-coding RNA GRASLND links melanoma differentiation and interferon-gamma response
Source: Front Mol Biosci. 2024 Sep 27;11:1471100. doi: 10.3389/fmolb.2024.1471100 (PMC11466874; doi:10.3389/fmolb.2024.1471100)
Supplement: Supplementary file 2 [file Table4.pdf]

Table S4: *Significance testing of GRASLND RNA expression was performed across nine melanoma cell lines (see Figure 1A). The mean differences in GRASLND expression measured by RT-qPCR were calculated from three biological replicates for each cell line, normalized to melanoma cell line A375 and compared against all other cell lines using a two-sided unpaired t-test. p-values marked in red indicate statistical significance, with a threshold of p<0.05.*

Cell Line 1	Cell Line 2	p-value	Mean Cell Line 1	Mean Cell Line 2	t-Statistic
501-mel	SK-MEL-239	0.00970	6.978	2.062	4.645
501-mel	Ma-Mel-86c	0.00517	6.978	1.630	5.547
501-mel	Ma-Mel-61a	0.00435	6.978	1.363	5.818
501-mel	SK-MEL-147	0.00247	6.978	0.418	6.777
501-mel	C8161	0.00197	6.978	0.044	7.200
501-mel	WM1361a	0.00196	6.978	0.036	7.209
501-mel	Ma-Mel-86a	0.00195	6.978	0.026	7.221
SK-MEL-239	Ma-Mel-86c	0.38569	2.062	1.630	0.973
SK-MEL-239	Ma-Mel-61a	0.19243	2.062	1.363	1.566
SK-MEL-239	SK-MEL-147	0.02207	2.062	0.418	3.635
SK-MEL-239	C8161	0.01027	2.062	0.044	4.569
SK-MEL-239	WM1361a	0.01012	2.062	0.036	4.588
SK-MEL-239	Ma-Mel-86a	0.00990	2.062	0.026	4.617
Ma-Mel-86c	Ma-Mel-61a	0.04752	1.630	1.363	2.826
Ma-Mel-86c	SK-MEL-147	0.00053	1.630	0.418	10.141
Ma-Mel-86c	C8161	0.00002	1.630	0.044	22.811
Ma-Mel-86c	WM1361a	0.00002	1.630	0.036	23.521
Ma-Mel-86c	Ma-Mel-86a	0.00002	1.630	0.026	24.974
Ma-Mel-61a	SK-MEL-147	0.00173	1.363	0.418	7.459
Ma-Mel-61a	C8161	0.00008	1.363	0.044	16.224
Ma-Mel-61a	WM1361a	0.00008	1.363	0.036	16.626
Ma-Mel-61a	Ma-Mel-86a	0.00006	1.363	0.026	17.403
SK-MEL-147	C8161	0.02686	0.418	0.044	3.417
SK-MEL-147	WM1361a	0.02446	0.418	0.036	3.520
SK-MEL-147	Ma-Mel-86a	0.02105	0.418	0.026	3.688
C8161	WM1361a	0.88638	0.044	0.036	0.152
C8161	Ma-Mel-86a	0.70266	0.044	0.026	0.410
WM1361a	Ma-Mel-86a	0.80940	0.036	0.026	0.258
