# Supplementary material for: Long non-coding RNA GRASLND links melanoma differentiation and interferon-gamma response
Source: Front Mol Biosci. 2024 Sep 27;11:1471100. doi: 10.3389/fmolb.2024.1471100 (PMC11466874; doi:10.3389/fmolb.2024.1471100)
Supplement: Supplementary file 3 [file Table1.pdf]

Table S1: *Primer list and sequences for RT-qPCR*

**Primer Name**	**Sequence (5′–3′)**
GRASLND-Fwd	aggattcaggggatgcacag
GRASLND-Rev	tgggctgaagatgagacgtt
GAPDH-Fwd	agccacatcgctcagacac
GAPDH-Rev	gcccaatacgaccaaatcc
HPRT1-Fwd	gaccagtcaacaggggacat
HPRT1-Rev	gtgtcaattatatcttccacaatcaa
MLANA-Fwd	gccactcttacaccacggct
MLANA-Rev	cagtaagactcccaggatcactactgtc
MALAT1-Fwd	gaaggaaggagcgctaacga
MALAT1-Rev	taccaaccactcgctttccc
PSMB9-Fwd	atgtctcccgaggagtgcag
PSMB9-Rev	gtccacaccggcagctgtaa
TAP1-Fwd	tccggaaaccgtgtctactt
TAP1-Rev	tcagggctttcgtacaggag
STAT1-Fwd	ttcacccttctagacttcagacc
STAT1-Rev	aggaacagagtagcaggaggga
