# Supplementary material for: Long non-coding RNA GRASLND links melanoma differentiation and interferon-gamma response
Source: Front Mol Biosci. 2024 Sep 27;11:1471100. doi: 10.3389/fmolb.2024.1471100 (PMC11466874; doi:10.3389/fmolb.2024.1471100)
Supplement: Supplementary file 4 [file Table2.pdf]

Table S2: *Detailed quality control information of RNA sequencing samples.*

Sample	Sample description	Total reads (millions)	Depth/Coverage	Mapping rate (% uniquely mapped reads)
lacZR1	shCtr, dox-induced, Rep1	41,8	2,77	85,99
lacZR2	shCtr, dox-induced, Rep2	35,1	2,32	83,47
lacZR3	shCtr, induced, Rep3	38,4	2,54	83,52
lacZ_ifnDoxR1	shCtr, IFNγ-treated, dox-induced, Rep1	39,3	2,59	82,98
lacZ_ifnR1	shCtr, IFNγ-treated, uninduced, Rep1	41,1	2,70	82,65
lacZ_ifnR2	shCtr, IFNγ-treated, uninduced, Rep2	42,3	2,79	82,9
lacz_ifnDoxR2	shCtr, IFNγ-treated, dox-induced, Rep2	37,2	2,46	84,04
sh4R1	shGRAS1, dox-induced, Rep1	34,8	2,30	84,99
sh4R2	shGRAS1, dox-induced, Rep2	48,6	3,21	84,91
sh4_ifnDoxR1	shGRAS1, IFNγ-treated, dox-induced, Rep1	34,0	2,25	81,28
sh4_ifnDoxR2	shGRAS1, IFNγ-treated, dox-induced, Rep2	37,6	2,48	83,51
sh4_ifnR1	shGRAS1, IFNγ-treated, uninduced, Rep1	38,1	2,52	82,69
sh4_ifnR2	shGRAS1, IFNγ-treated, uninduced, Rep2	46,3	3,05	83,09
sh8R1	shGRAS1, dox-induced, Rep1	33,1	2,18	85,8
sh8R2	shGRAS1, dox-induced, Rep2	40,7	2,69	85,08
sh8_ifnDoxR1	shGRAS2, IFNγ-treated, dox-induced, Rep1	27,8	1,84	84,57
sh8_ifnDoxR2	shGRAS2, IFNγ-treated, dox-induced, Rep2	35,2	2,34	82,65
sh8_ifnR1	shGRAS2, IFNγ-treated, uninduced, Rep1	40,7	2,69	82,14
sh8_ifnR2	shGRAS2, IFNγ-treated, uninduced, Rep2	57,6	3,82	86,28
