# Supplementary material for: Long non-coding RNA GRASLND links melanoma differentiation and interferon-gamma response
Source: Front Mol Biosci. 2024 Sep 27;11:1471100. doi: 10.3389/fmolb.2024.1471100 (PMC11466874; doi:10.3389/fmolb.2024.1471100)
Supplement: Supplementary file 5 [file Table6.pdf]

Table S6: *Significance testing of AXL protein expression was performed across nine melanoma cell lines (see Figure 1B, bottom). The mean differences in AXL protein expression measured by western blotting, were calculated from three biological replicates for each cell line, normalized to melanoma cell line A375 and compared against all other cell lines using a two-sided unpaired t-test. p-values marked in red indicate statistical significance, with a threshold of $p<0.05$.*

Cell Line 1	Cell Line 2	p-value	Mean Cell Line 1	Mean Cell Line 2	t-Statistic
501-mel	SK-MEL-239	0.76942	0.071	0.058	0.314
501-mel	Ma-Mel-86c	0.47879	0.071	0.043	0.780
501-mel	Ma-Mel-61a	0.67810	0.071	0.055	0.447
501-mel	SK-MEL-147	0.00118	0.071	6.282	-8.246
501-mel	C8161	0.00106	0.071	6.710	-8.481
501-mel	WM1361a	0.00002	0.071	4.001	-22.459
501-mel	Ma-Mel-86a	0.02021	0.071	3.550	-3.735
SK-MEL-239	Ma-Mel-86c	0.63417	0.058	0.043	0.514
SK-MEL-239	Ma-Mel-61a	0.92572	0.058	0.055	0.099
SK-MEL-239	SK-MEL-147	0.00117	0.058	6.282	-8.267
SK-MEL-239	C8161	0.00105	0.058	6.710	-8.501
SK-MEL-239	WM1361a	0.00002	0.058	4.001	-22.706
SK-MEL-239	Ma-Mel-86a	0.01995	0.058	3.550	-3.750
Ma-Mel-86c	Ma-Mel-61a	0.55166	0.043	0.055	-0.649
Ma-Mel-86c	SK-MEL-147	0.00116	0.043	6.282	-8.290
Ma-Mel-86c	C8161	0.00104	0.043	6.710	-8.523
Ma-Mel-86c	WM1361a	0.00002	0.043	4.001	-22.980
Ma-Mel-86c	Ma-Mel-86a	0.01966	0.043	3.550	-3.767
Ma-Mel-61a	SK-MEL-147	0.00116	0.055	6.282	-8.275
Ma-Mel-61a	C8161	0.00105	0.055	6.710	-8.508
Ma-Mel-61a	WM1361a	0.00002	0.055	4.001	-22.904
Ma-Mel-61a	Ma-Mel-86a	0.01988	0.055	3.550	-3.754
SK-MEL-147	C8161	0.71319	6.282	6.710	-0.395
SK-MEL-147	WM1361a	0.04173	6.282	4.001	2.956
SK-MEL-147	Ma-Mel-86a	0.08456	6.282	3.550	2.282
C8161	WM1361a	0.02769	6.710	4.001	3.384
C8161	Ma-Mel-86a	0.06010	6.710	3.550	2.599
WM1361a	Ma-Mel-86a	0.65875	4.001	3.550	0.476
