# Supplementary material for: Long non-coding RNA GRASLND links melanoma differentiation and interferon-gamma response
Source: Front Mol Biosci. 2024 Sep 27;11:1471100. doi: 10.3389/fmolb.2024.1471100 (PMC11466874; doi:10.3389/fmolb.2024.1471100)
Supplement: Supplementary file 6 [file Image2.pdf]

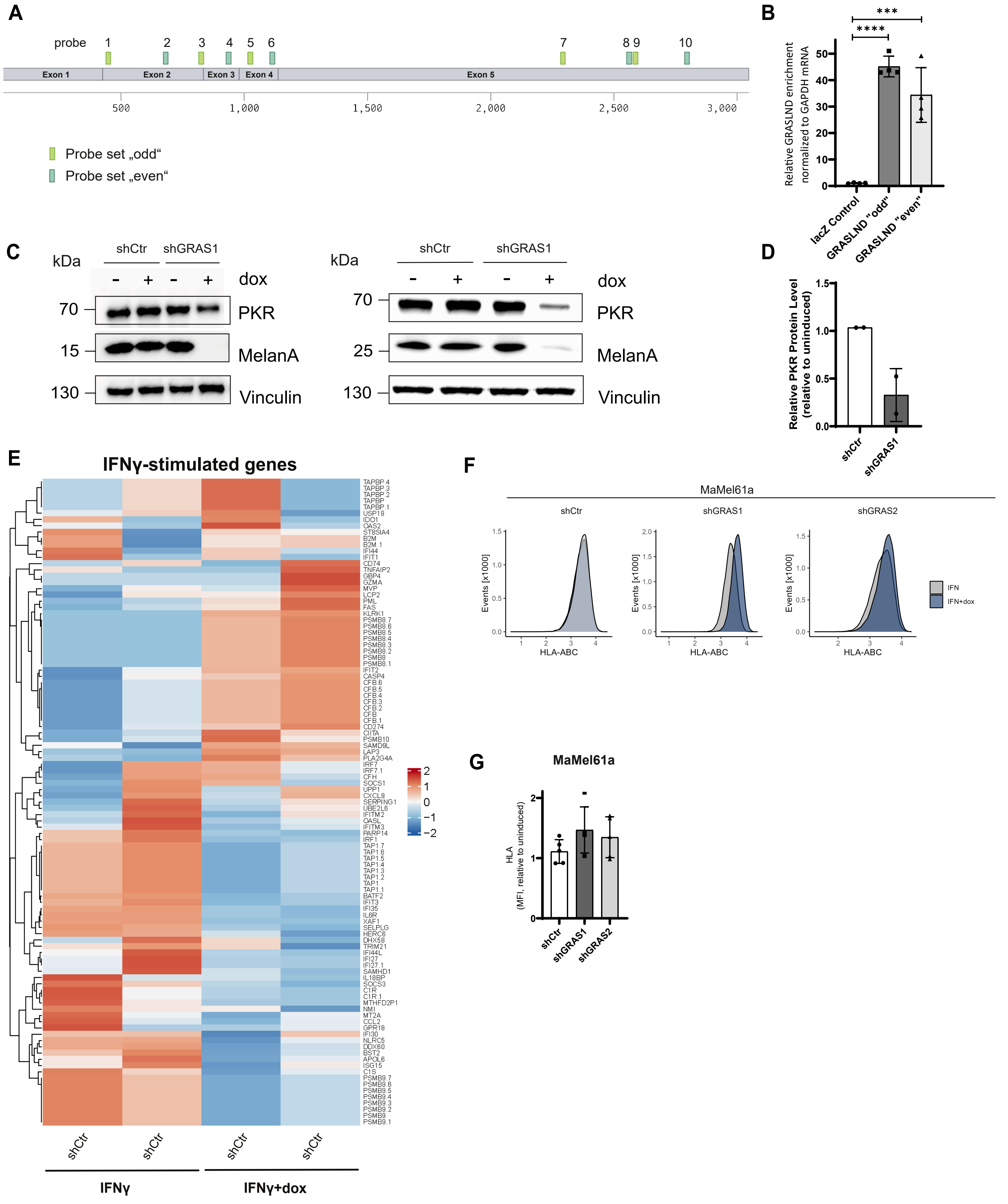

Figure S2. GRASLND impairs IFNγ signalling. **(A)** Schematic overview of biotinylated DNA probe sets used in GRASLND RNA pulldown experiment. **(B)** GRASLND RNA pulldown using biotinylated DNA probes. GRASLND enrichment was validated by RT-qPCR. n=4 independent biological replicates. **(C, D)** Expression of PKR after GRASLND knockdown in 501-mel cells induced with doxycycline (2 µg/mL) for 72 hours analyzed by Western blotting, Vinculin served as loading control. Quantification of PKR levels given as mean±SD. n=2 independent biological replicates. **(E)** Heatmap of differentially expressed IFNγ-stimulated genes after IFNγ treatment and doxycycline induction (2 µg/mL) for 6 days of 501Mel control shRNA (shCtr) cells using RNA-sequencing. **(F)** HLA-I surface expression in MaMel61a cells determined by flow cytometry. Representative histograms of control and GRASLND knockdown cell lines from independent biological replicates (n=5). **(E)** Fold change of MFI of IFNγ-only to IFNγ +dox treated cells given as mean±SD in MaMel61a cells.
