# Supplementary material for: Long non-coding RNA GRASLND links melanoma differentiation and interferon-gamma response
Source: Front Mol Biosci. 2024 Sep 27;11:1471100. doi: 10.3389/fmolb.2024.1471100 (PMC11466874; doi:10.3389/fmolb.2024.1471100)

Supplementary Figure S3
Uncropped Blots

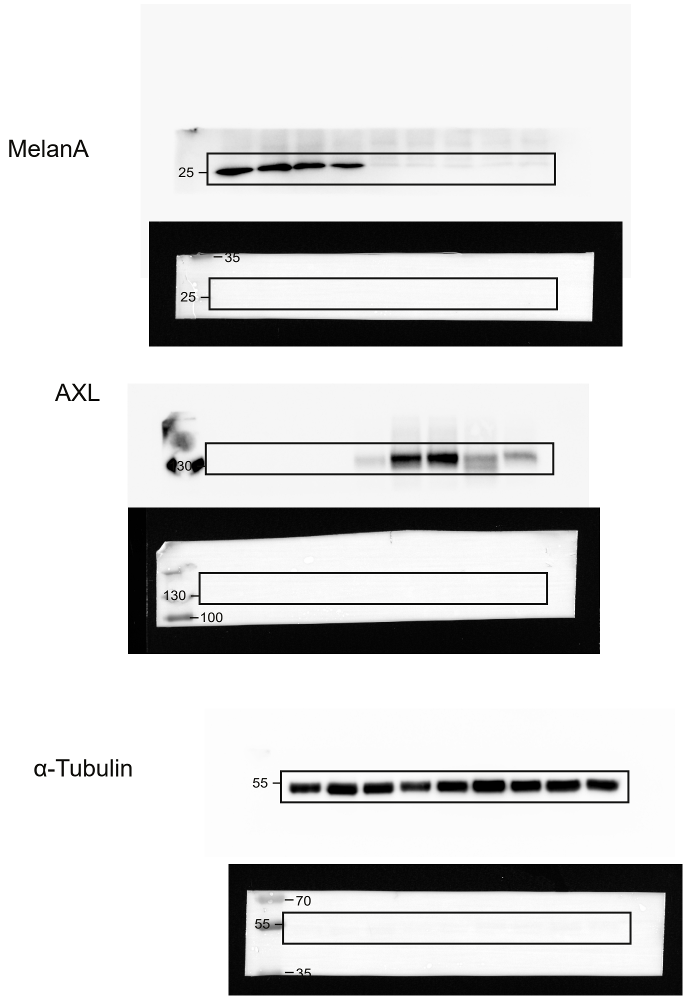

Figure 1B - Biological Triplicates

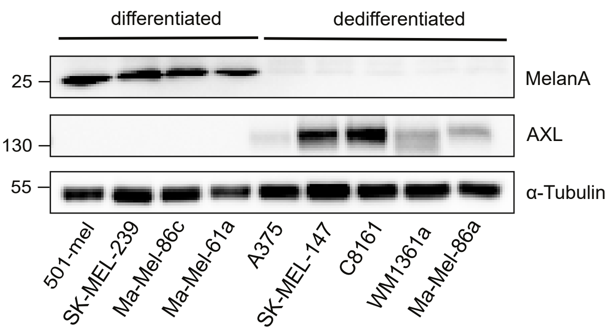

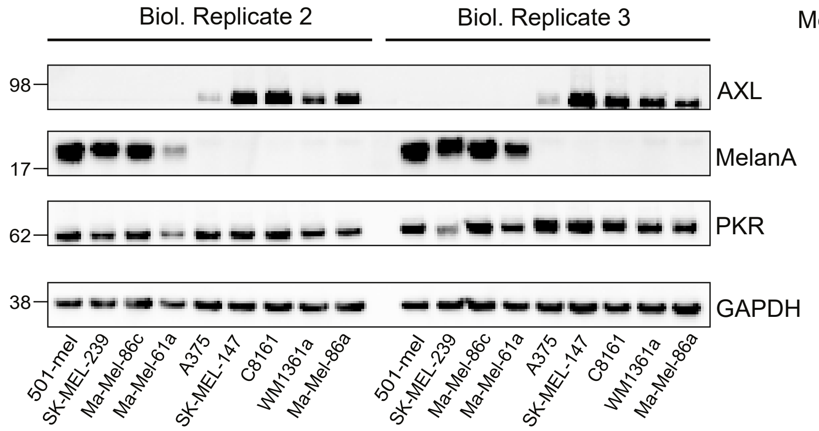

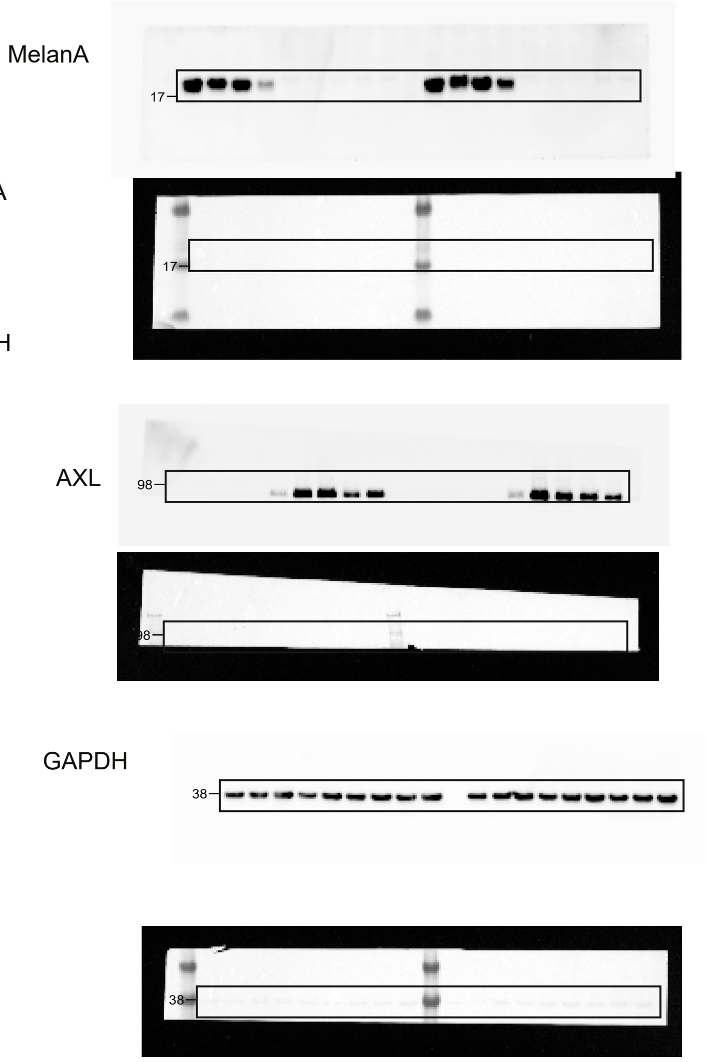

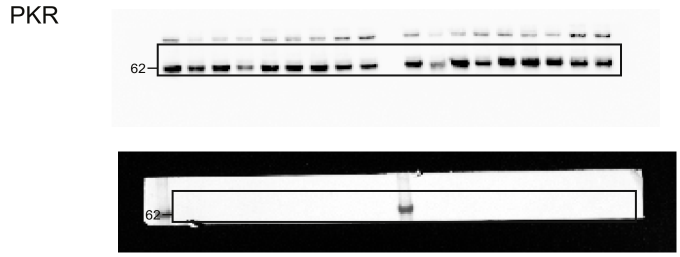

Uncropped Blots

Figure 2C - Biological Triplicates

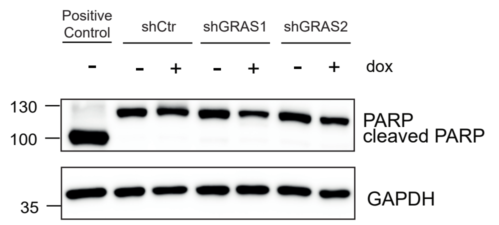

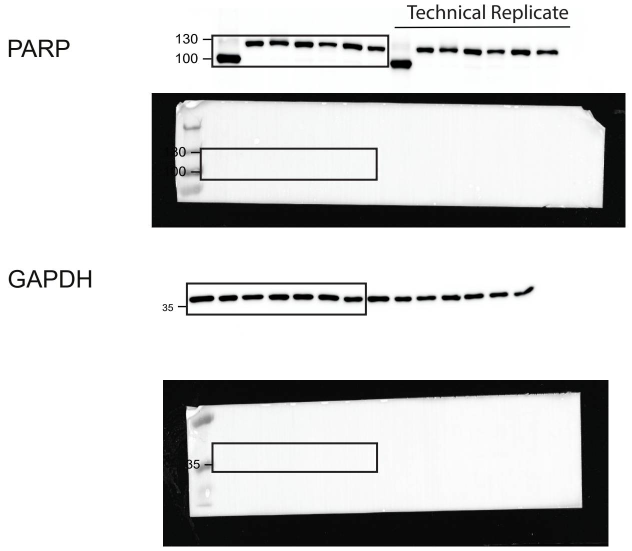

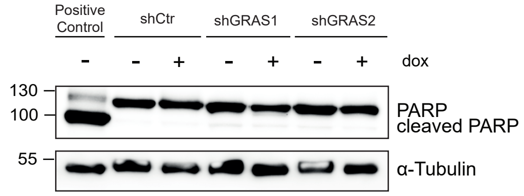

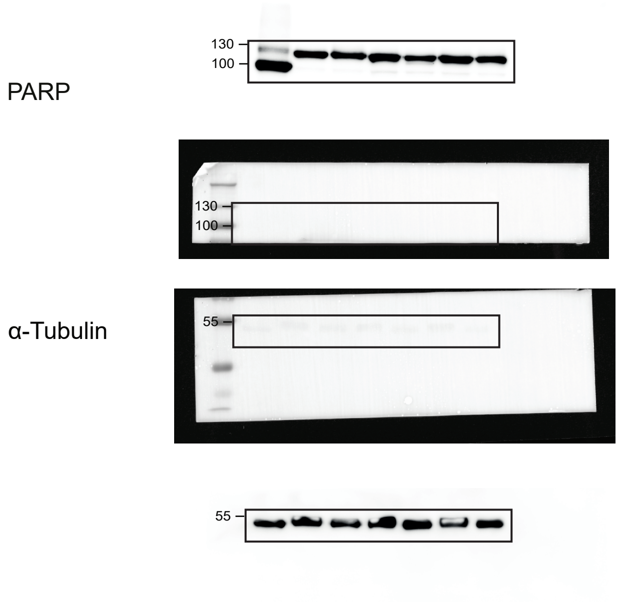

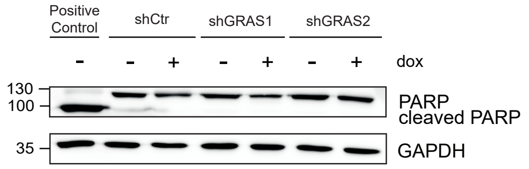

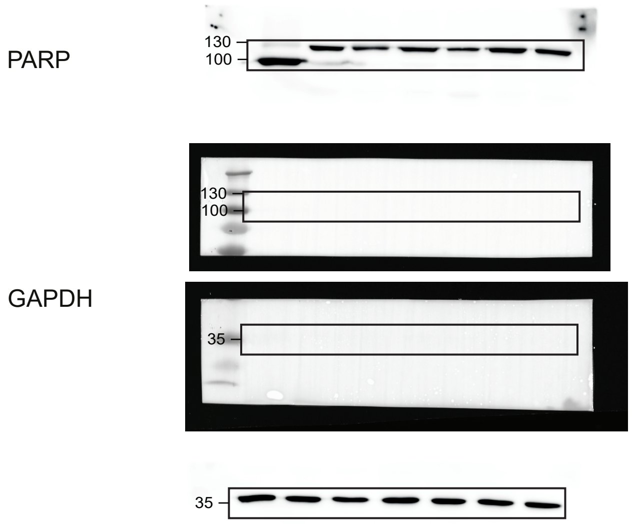

Uncropped Blots

Figure 3A (top) - Biological Triplicates

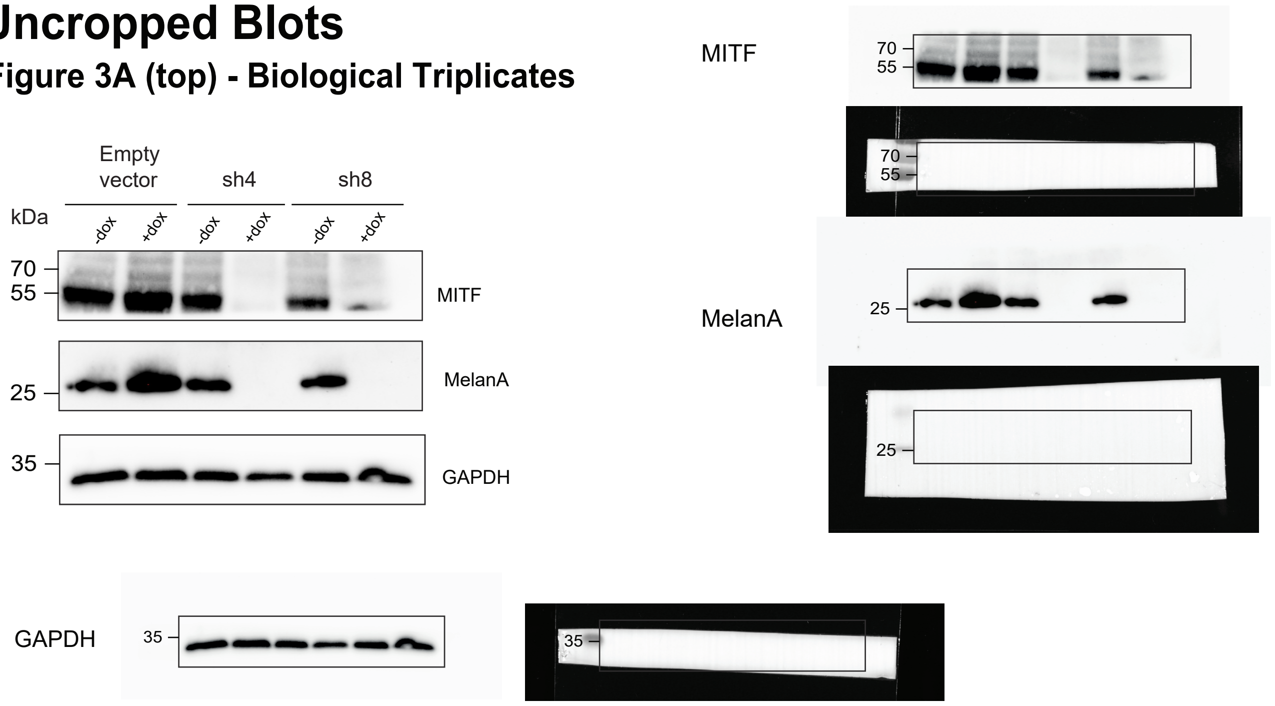

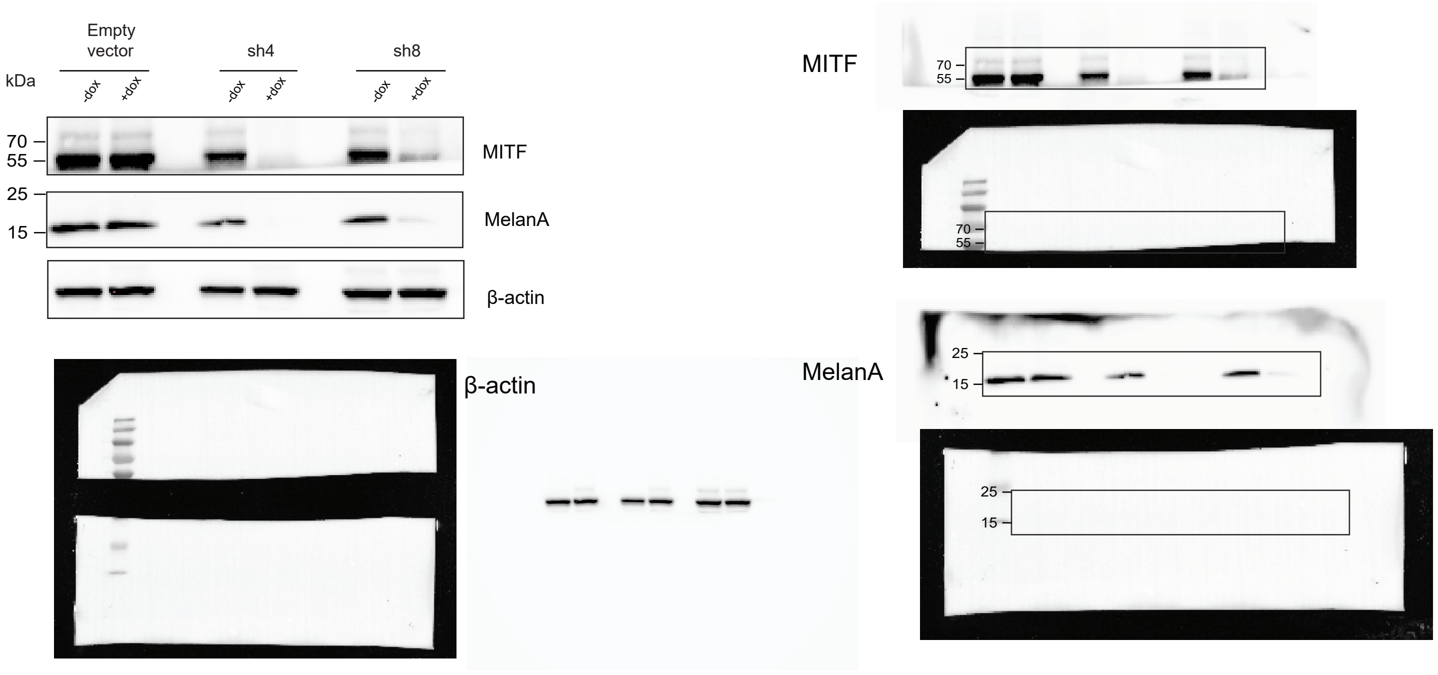

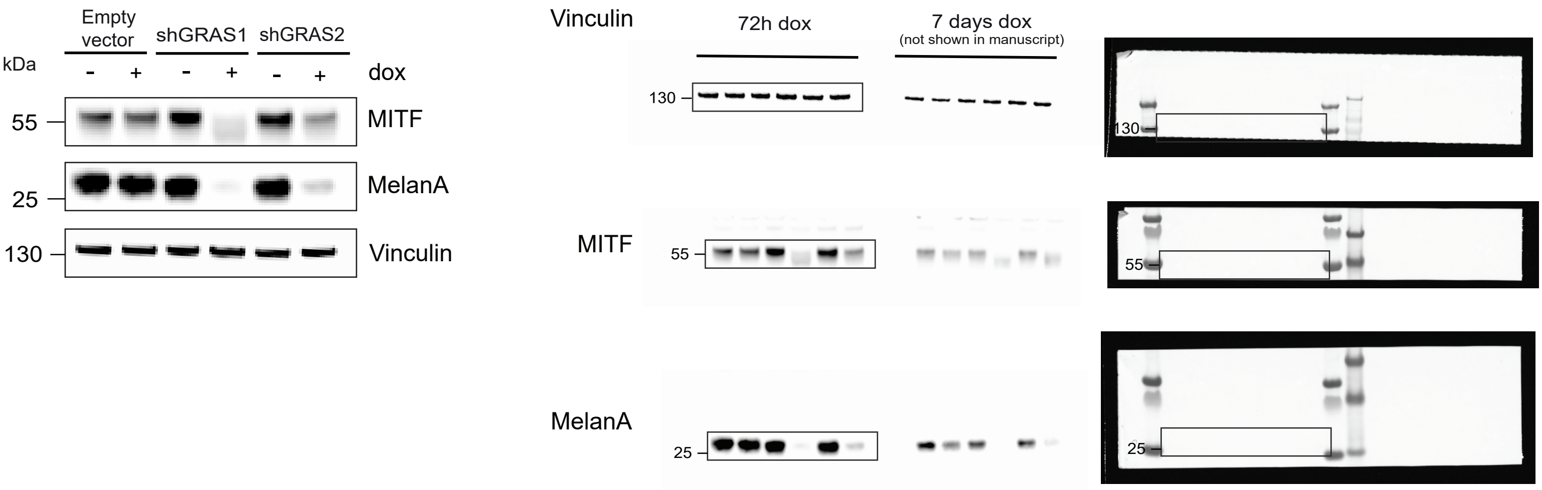

Uncropped Blots

Figure 3A (bottom) - Biological Triplicates

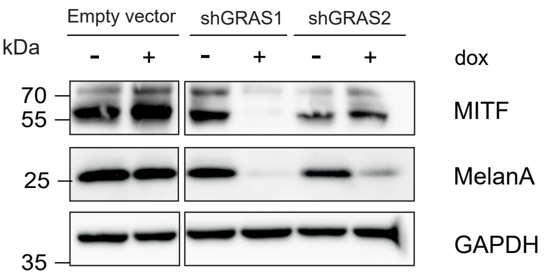

Non-targeting (NT) shRNA control removed for the manuscript.

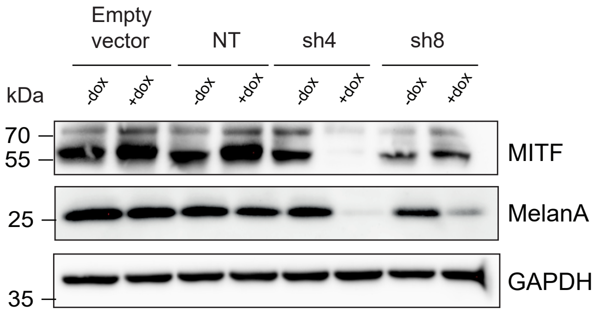

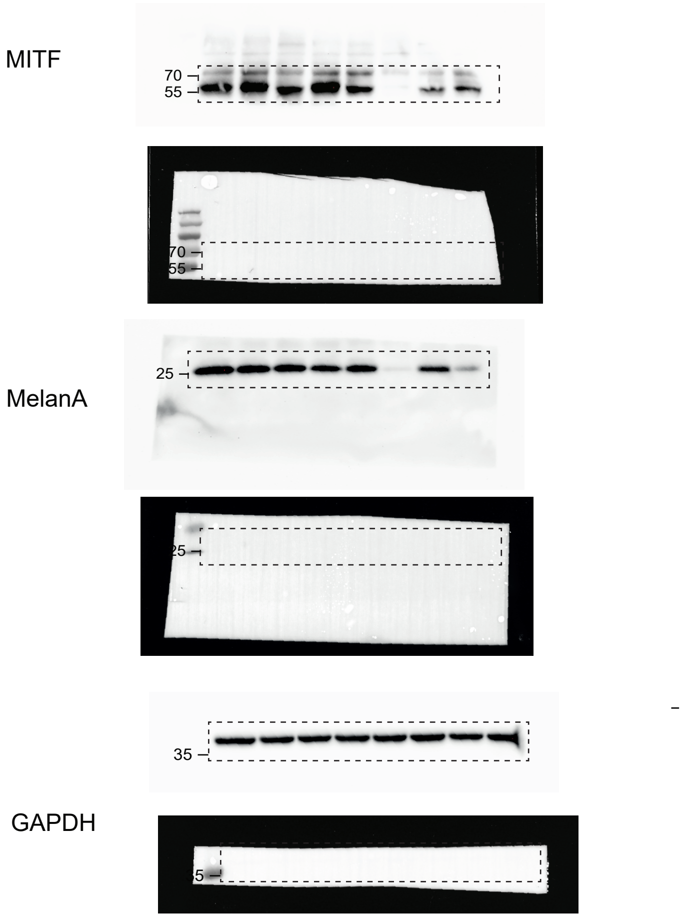

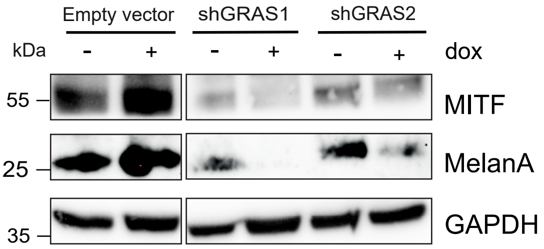

Non-targeting (NT) shRNA control removed for the manuscript.

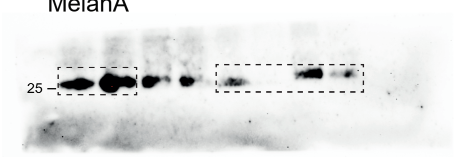

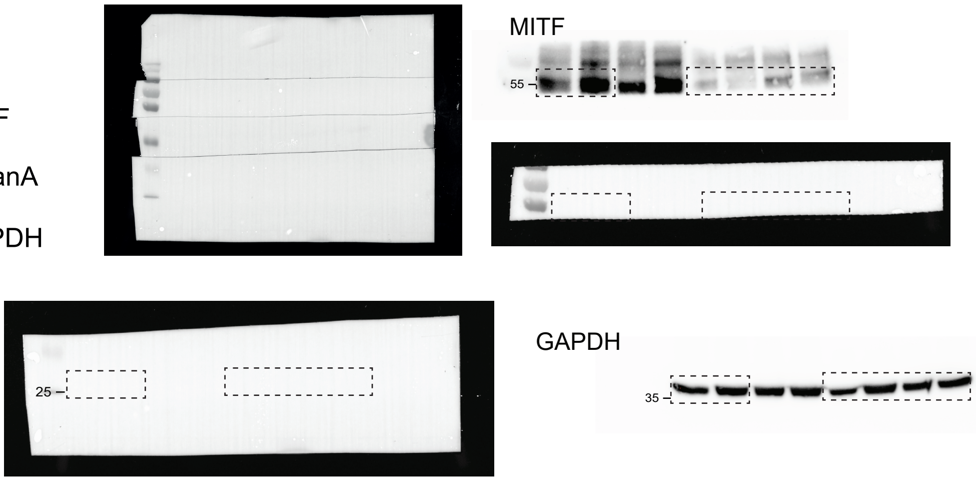

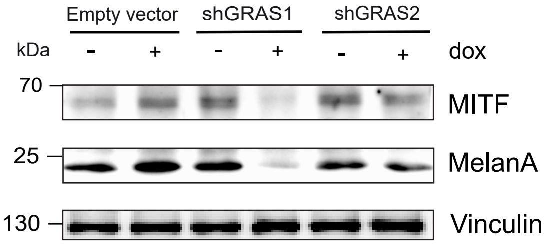

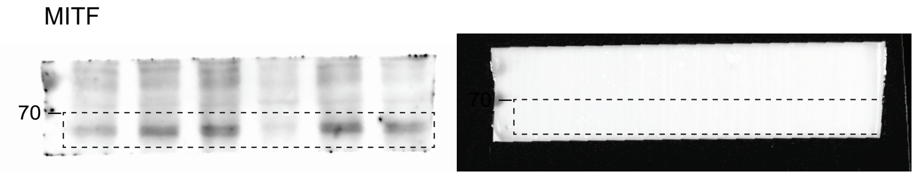

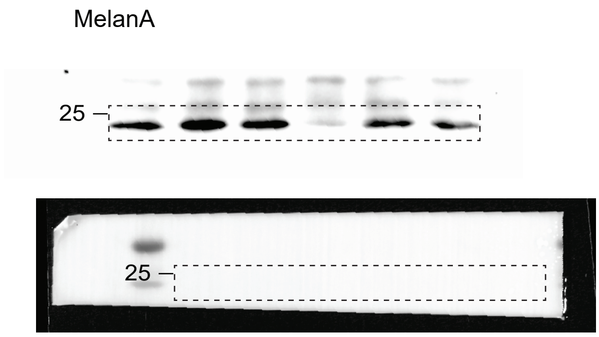

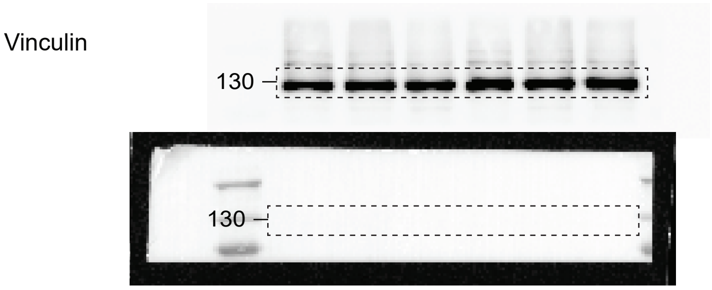

Uncropped Blots

Figure 5A - Biological Triplicates

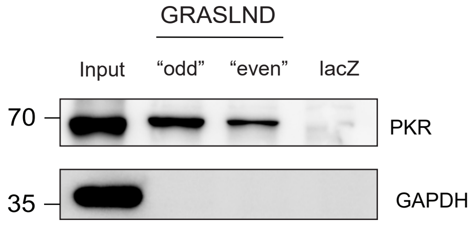

PKR

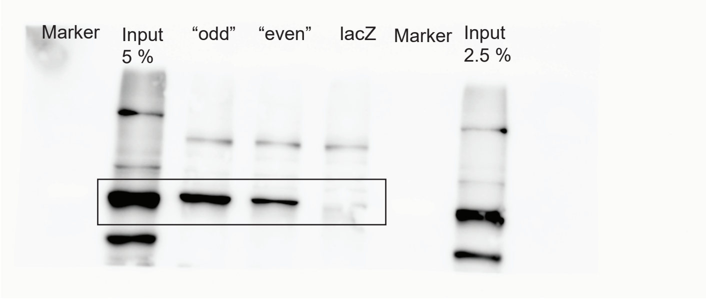

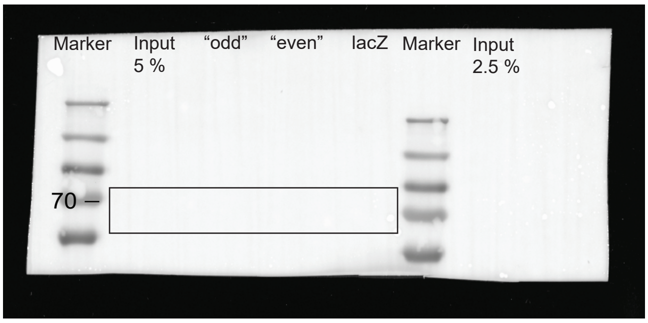

GAPDH

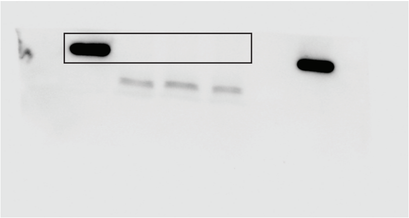

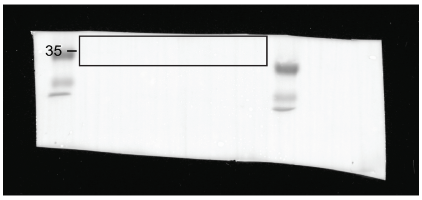

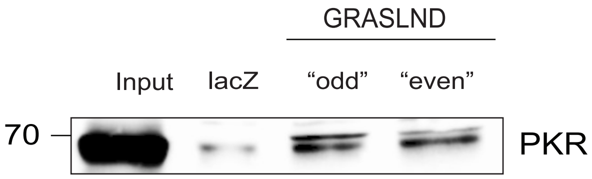

PKR

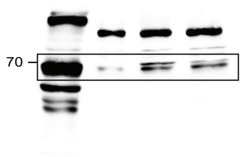

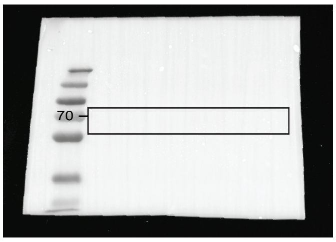

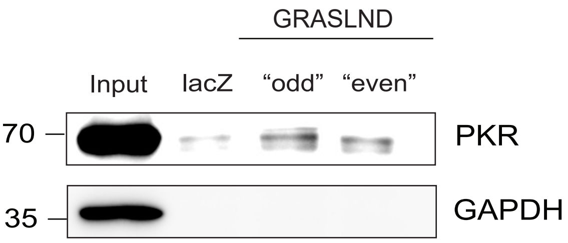

PKR

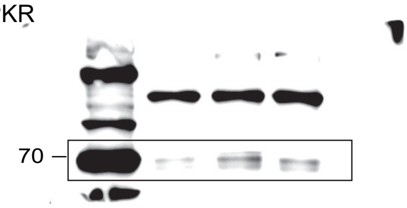

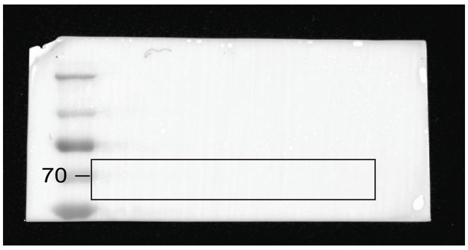

GAPDH

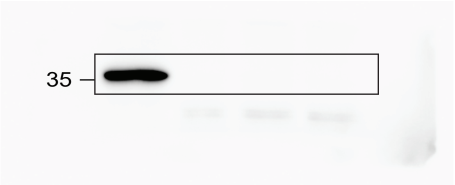

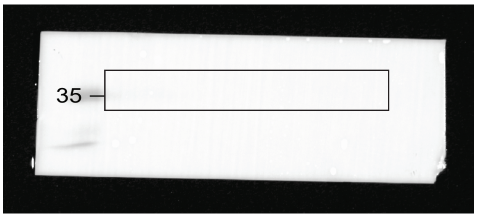

Uncropped Blots

Supplementary Figure S2C

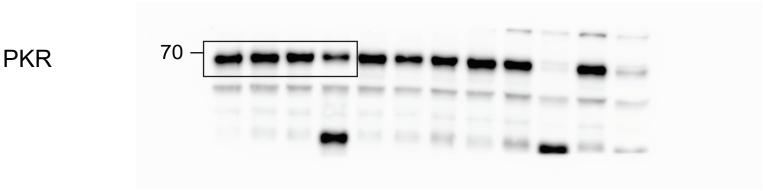

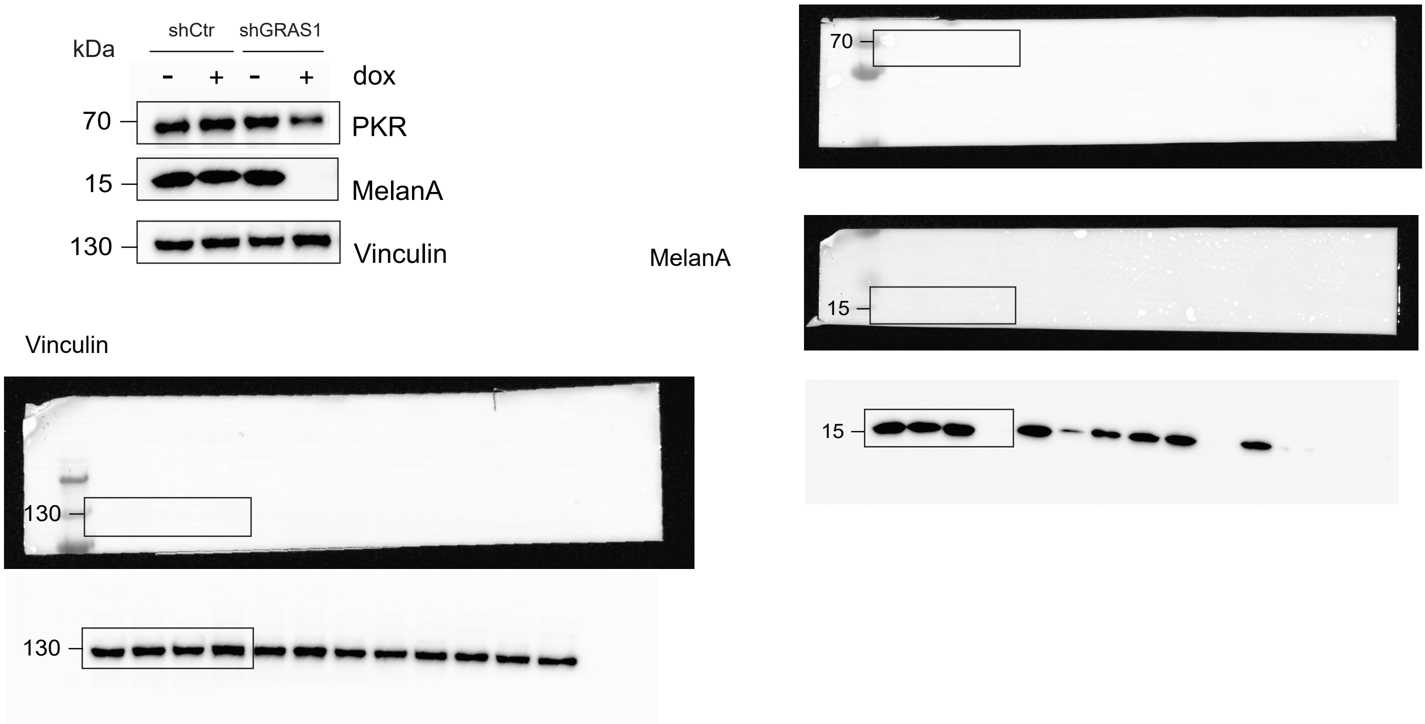

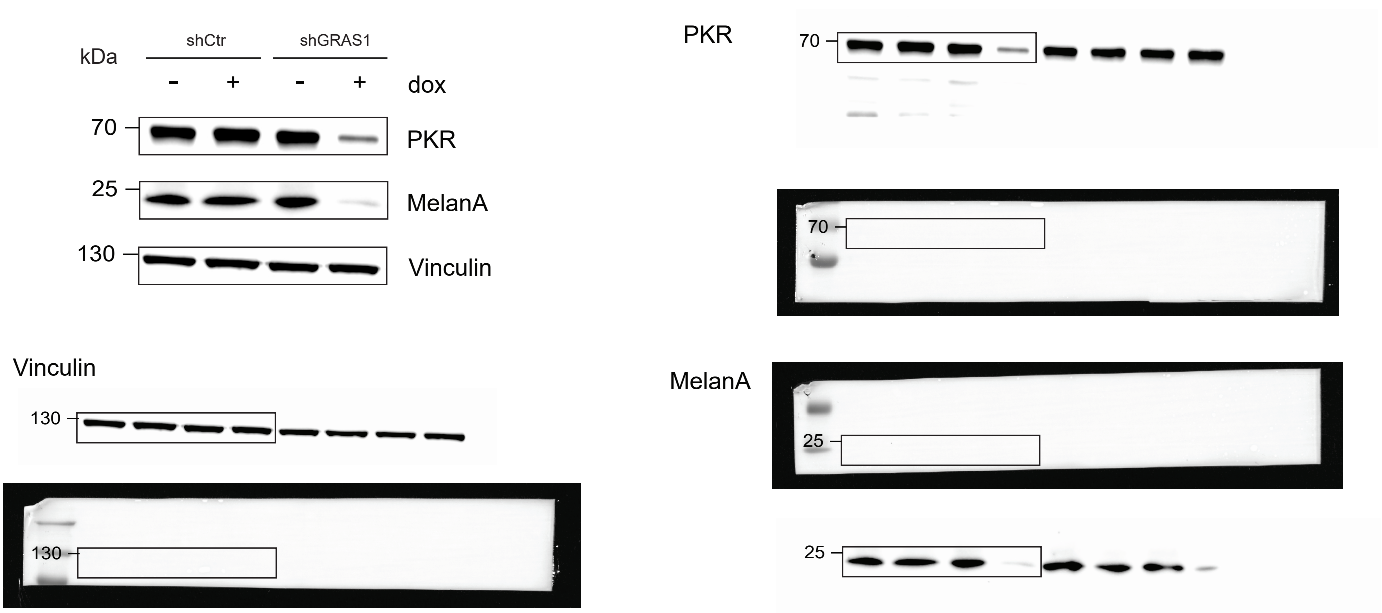

Supplementary Figure S1B

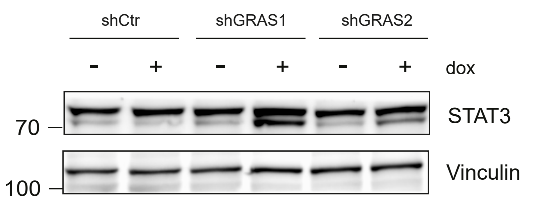

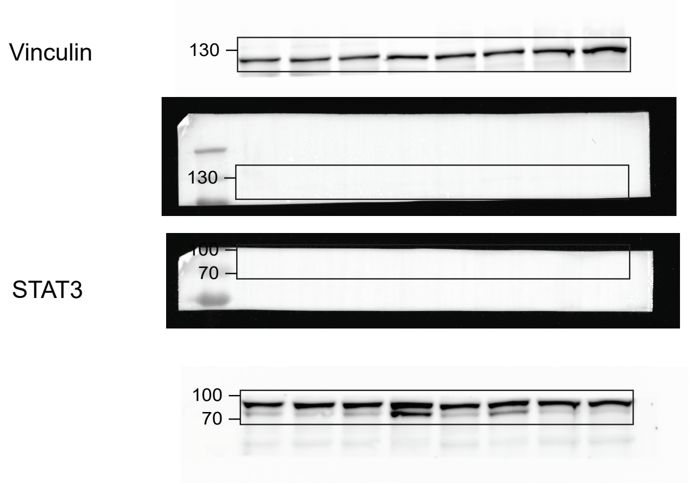

Supplement: Supplementary file 7 [file Image3.pdf]
