# Supplementary material for: Long non-coding RNA GRASLND links melanoma differentiation and interferon-gamma response
Source: Front Mol Biosci. 2024 Sep 27;11:1471100. doi: 10.3389/fmolb.2024.1471100 (PMC11466874; doi:10.3389/fmolb.2024.1471100)
Supplement: Supplementary file 8 [file Table3.pdf]

Table S3: *Biotinylated oligonucleotides list and sequences for RNA pulldown.*

**Probe**	**Sequence (5′–3′)**
Odd, probe 1	tgtaggaatcaggggagtgt
Odd, probe 2	gcttcgcagatcttagattc
Odd, probe 3	agacgttacattccacattc
Odd, probe 4	atgagtagtcaccttccatg
Odd, probe 5	agaatctacggagcttgcat
Even, probe 1	ggtgaacagacagactttcc
Even, probe 2	gtactcaaccaggaacttct
Even, probe 3	ctctggcaggaaagtcttgt
Even, probe 4	catgcatagaagatccggtt
Even, probe 5	ccaagggccatattcaattg
lacZ Control, probe 1	aatgtgagcgagtaacaacc
lacZ Control, probe 2	attaagttgggtaacgccag
lacZ Control, probe 3	aataattcgcgtctggcctt
lacZ Control, probe 4	aattcagacggcaaacgct
lacZ Control, probe 5	atcttccagataactgccgt
