# Supplementary material for: Long non-coding RNA GRASLND links melanoma differentiation and interferon-gamma response
Source: Front Mol Biosci. 2024 Sep 27;11:1471100. doi: 10.3389/fmolb.2024.1471100 (PMC11466874; doi:10.3389/fmolb.2024.1471100)
Supplement: Supplementary file 9 [file Table7.pdf]

Table S7: *List of downregulated genes from HALLMARK pathways obtained from GSEA of RNA sequencing results after GRASLND knockdown in 501Mel cells. Striking genes related to observed phenotypes are highlighted in red.*

**HALLMARK Pathway**	**Downregulated genes**
HALLMARK_ESTROGEN_RESPONSE_EARLY	RBBP8, CCND1, FDFT1, SLC22A5, RRP12, RAB17, KAZN, MYC, UNC119, BAG1, NCOR2, SLC16A1, SVIL, NAV2, DHCR7, PPIF, MYBBP1A, SCARB1, FKBP4, ITPK1, NXT1, SLC7A2, FRK, FLNB, FASN, INPP5F, SLC1A4, ADCY1, FOXC1, ABLIM1, HES1, FHL2, SLC7A5, SLC26A2, GREB1
HALLMARK_ESTROGEN_RESPONSE_LATE	RBBP8, RNASEH2A, MOCS2, CCND1, FDFT1, IMPA2, SLC22A5, GALE, BAG1, NCOR2, SGK1, SLC16A1, ISG20, DHCR7, PPIF, SCARB1, GINS2, FKBP4, ITPK1, SORD, NXT1, UGDH, XRCC3, ST6GALNAC2, FRK, CDC6, FLNB, SLC1A4, FOXC1, CKB, SLC7A5, SLC26A2
HALLMARK_DNA_REPAIR	PDE4B, CSTF3, ERCC3, TAF13, SMAD5, POLR1D, ADRM1, POLR2C, POLR1C, POLR2K, NT5C3A, MRPL40, UPF3B, POLR2G, GTF2H3, IMPDH2, SNAPC4, POLA1, POLB, SNAPC5, ERCC2, RFC5, POLR2H, APRT, POLR2J, SF3A3, PNP, SAC3D1, LIG1, POLR2E, POLD1, ADA, RNMT, RPA2, POLA2, RFC2, RAE1, NUDT21, NCBP2, NME1, ZWINT, TYMS, REV3L, POLR2F, TAF12, ALYREF, TMED2, POLR2D, SSRP1, UMPS, RAD51, GTF2H1, FEN1, PCNA, RALA, PRIM1, GTF2A2, RFC3, RFC4
HALLMARK_FATTY_ACID_METABOLISM	UROD, IDH3B, CRYZ, PDHA1, HCCS, HPGD, MDH2, CPOX, DHCR24, HADH, HSPH1, SDHD, MIF, METAP1, ACO2, HMGCS1, ERP29, ADSL, ACAT2, UROS, APEX1, ME1, NTHL1, ECHS1, SLC22A5, CBR1, ALDH9A1, ALDOA, SMS, IDI1, GCDH, HSP90AA1, MDH1, ACSL1, RDH11, FH, ECI1, HSDL2, PRDX6, SUCLG1, UGDH, GLUL, MIX23, FASN, IDH1, LDHA, ODC1, H2AZ1
HALLMARK_UV_RESPONSE_UP	SPR, POLR2H, SLC6A8, CHKA, PDAP1, CASP3, GRPEL1, RAB27A, CNP, E2F5, EIF5, TARS1, IGFBP2, TGFBRAP1, ALDOA, ARRB2, HNRNPU, SIGMAR1, PPIF, DDX21, NUP58, ATP6V1C1, CTSV, AMD1, SOD2, H2AX, FKBP4, FEN1, CCNE1, CEBPG, STIP1, EIF2S3, ASNS, RFC4, TFRC, CHRNA5, PPAT, **CDK2**
HALLMARK_CHOLESTEROL_HOMEOSTASIS	ACTG1, HMGCS1, ACAT2, ERRFI1, CHKA, HMGCR, FDFT1, CBS, MVD, CYP51A1, FDPS, IDI1, LGMN, SQLE, DHCR7, ANXA5, PLSCR1, FASN, TMEM97, LGALS3, SCD, JAG1
HALLMARK_GLYCOLYSIS	AK4, MDH2, HMMR, MIF, RARS1, VEGFA, CENPA, CLN6, HSPA5, ERO1A, SLC25A13, ME1, TPI1, CTH, GLCE, AKR1A1, PPIA, PYGB, GOT2, GALE, CYB5A, CACNA1H, TXN, RRAGD, ALDH9A1, SDC3,

	ALDOA, CHST6, AURKA, MDH1, ABCB6, PYGL, ISG20, SLC37A4, **CDK1**, GFPT1, ENO1, SLC25A10, POLR3K, PRPS1, PAXIP1, FKBP4, ME2, RPE, B4GALT2, DEPDC1, IDH1, MERTK, HK2, LDHA, MXI1, MET, HOMER1
HALLMARK_REACTIVE_OXYGEN_SPECIES_PATHWAY	ATOX1, NQO1, NDUFB4, PDLIM1, SOD1, HMOX2, FTL, GCLM, ERCC2, PRDX1, TXN, SRXN1, HHEX, SBNO2, PRDX6, TXNRD1, MBP, PRDX4, SOD2, ABCC1, GSR
HALLMARK_UNFOLDED_PROTEIN_RESPONSE	EEF2, EIF4A1, CNOT6, VEGFA, RPS14, HSPA5, EIF2S1, LSM4, FUS, ERO1A, EIF4A2, KHSRP, PDIA6, GEMIN4, TARS1, EXOSC9, SDAD1, MTREX, HSPA9, SPCS1, EXOSC4, XPOT, NOP14, EIF4E, IARS1, PARN, NHP2, EXOSC10, RRP9, NPM1, NOP56, H2AX, HSP90B1, ALDH18A1, SERP1, CEBPG, EXOSC2, PSAT1, DKC1, EIF4G1, ATF4, ASNS, SLC1A4, MTHFD2, EIF4A3, EIF4EBP1, NOLC1, SLC7A5
HALLMARK_MTORC1_SIGNALING	GPI, PSMD14, HSPA5, PSMA4, HMGCS1, NUFIP1, NMT1, ERO1A, DDIT3, ME1, PPA1, BUB1, TPI1, PRDX1, HMGCR, PNP, PDAP1, WARS1, PLK1, ABCF2, CTH, CANX, PPIA, PSME3, CYP51A1, ALDOA, IMMT, SDF2L1, IDI1, PHGDH, AURKA, HSPA9, POLR3G, ELOVL6, GGA2, RDH11, EIF2S2, PSMD12, SLC37A4, LGMN, PNO1, SQLE, DHCR7, ACACA, NFKBIB, RRM2, ETF1, SHMT2, RRP9, TXNRD1, ENO1, TUBG1, HSPE1, HSPA4, GTF2H1, MCM2, HSP90B1, SERP1, SORD, HSPD1, DHFR, GMPS, UCHL5, PSAT1, STIP1, NUP205, UNG, IDH1, ACSL3, ASNS, HK2, SLC1A4, EEF1E1, LDHA, GSR, MCM4, TFRC, MTHFD2, CACYBP, TMEM97, TOMM40, PSPH, CCT6A, PSMG1, SCD, IFRD1, CDC25A, SLC7A5
HALLMARK_G2M_CHECKPOINT	KIF22, EZH2, BUB3, LBR, WRN, BIRC5, GSPT1, EWSR1, NASP, POLE, HMGN2, SMC1A, SMARCC1, MKI67, CKS1B, MTF2, E2F2, SMC2, CDC7, MEIS2, XPO1, H2AZ2, G3BP1, SNRPD1, CUL4A, HSPA8, HMMR, ORC6, RAD23B, HIRA, TENT4A, DBF4, CENPA, NSD2, TRA2B, TFDP1, CHEK1, PRIM2, E2F1, SS18, SRSF2, AURKB, SMC4, MCM6, BUB1, BRCA2, RBM14, NUP98, E2F3, PLK1, CCND1, SRSF1, CDKN3, LIG3, SLC12A2, POLQ, KPNA2, CHAF1A, **MYC**, CDC25B, RPA2, POLA2, PRMT5, SFPQ, DTYMK, TACC3, HUS1, KIF23, AURKA, HNRNPD, CKS2, UBE2S, SYNCRIP, HNRNPU, FBXO5, KPNB1, ORC5, SQLE, **CDK1**, LMNB1, UPF1, INCENP, **CDK4**, CCNA2, ESPL1, AMD1, CDC45, EXO1, PTTG1, H2AX, GINS2, MCM2, MCM3, H2BC12, MAD2L1, DKC1, TNPO2, HMGA1,

	CDC6, NCL, RAD54L, MYBL2, ODC1, NOLC1, H2AZ1, CDC25A, SLC7A5, SLC7A1
HALLMARK_OXIDATIVE_PHOSPHORYLATION	NDUFS7, NDUFV1, ATP5MC2, MRPS11, TIMM9, NDUFS3, NDUFB4, BAX, SDHA, PDHX, UQCRC1, IDH3B, HTRA2, ATP5F1B, PDHA1, NDUFC1, COX5B, COX15, ATP5ME, ACADSB, HCCS, NDUFB6, NDUFC2, MFN2, COX7B, ETFA, MDH2, SLC25A3, NDUFA4, SDHD, ATP5F1C, SLC25A4, ATP5PO, ATP5MC1, CS, UQCRQ, ACO2, COX4I1, GPI, ATP6V0C, NDUFV2, NDUFA8, NDUFAB1, UQCRH, GLUD1, OXA1L, COX7C, TOMM70, SLC25A6, COX11, UQCRC2, ECHS1, UQCRFS1, NDUFA7, GRPEL1, NDUFS6, FDX1, OPA1, SUPV3L1, ATP5MC3, COX5A, AIFM1, IDH3A, GOT2, CYB5A, SDHB, ATP5MF, FXN, COX7A2L, MTRR, OAT, SLC25A11, MTX2, IMMT, SLC25A5, MDH1, POLR2F, HSPA9, CYC1, TIMM17A, TOMM22, MRPL11, FH, MRPS12, ECI1, ACAT1, PRDX3, VDAC2, MRPS30, MRPS15, MRPL34, ATP5F1D, PMPCA, PHB2, ATP5F1A, VDAC1, ATP6V1C1, TIMM13, LRPPRC, SUCLG1, LDHB, MRPL35, AFG3L2, IDH1, CYCS, LDHA, DLAT, TIMM50
HALLMARK_E2F_TARGETS	BUB1B, KIF22, EZH2, CCP110, RFC1, CDCA8, LBR, SMC3, BIRC5, GSPT1, CNOT9, NASP, DCK, ZW10, POLE, DCLRE1B, SMC1A, DNMT1, TBRG4, MKI67, CKS1B, RPA1, MLH1, XPO1, DEK, EED, PMS2, HELLS, HMMR, ORC6, ORC2, PAICS, DSCC1, TRA2B, ANP32E, NUP153, CHEK1, EIF2S1, PRIM2, SRSF2, AURKB, SMC4, MCM6, BRCA2, RNASEH2A, RAD1, MRE11, PLK1, LIG1, CIT, SRSF1, DLGAP5, CDKN3, POLD1, MMS22L, USP1, GINS1, E2F8, XRCC6, EXOSC8, ASF1B, SLBP, KPNA2, MYC, PNN, CDC25B, RPA2, POLA2, SHMT1, AK2, RFC2, NUDT21, PHF5A, NME1, TACC3, HUS1, AURKA, HNRNPD, ATAD2, CKS2, UBE2S, KIF18B, SYNCRIP, IPO7, CENPM, SNRPB, DONSON, TK1, NAP1L1, CTPS1, RAN, GINS3, CDK1, LMNB1, MSH2, RRM2, CDK4, BRCA1, ESPL1, WEE1, PRKDC, TUBB, UBE2T, SSRP1, TUBG1, NUP107, NOP56, PTTG1, PRPS1, PRDX4, H2AX, POP7, MCM2, PCNA, CSE1L, CCNE1, DCTPP1, MELK, TIPIN, MCM3, MCM7, MAD2L1, RAD51AP1, PSMC3IP, DEPDC1, CHEK2, POLD2, NUP205, UNG, HMGA1, LYAR, GINS4, RFC3, MCM4, PA2G4, TFRC, MTHFD2, MYBL2, RANBP1, TRIP13, NOLC1, H2AZ1, CDC25A
HALLMARK_MYC_TARGETS_V2	TCOF1, DDX18, TFB2M, PES1, NOC4L, PLK1, RRP12, SUPV3L1, GRWD1, PPAN, MYC, RABEPK, PPRC1, NOP16, SLC19A1, PUS1, WDR74, UTP20, FARSA, GNL3, IMP4, BYSL, MYBBP1A, RCL1, CDK4, AIMP2, RRP9, NPM1, WDR43, SLC29A2, NOP56, HSPE1, IPO4, DCTPP1, SORD, NOP2,

	HSPD1, PRMT3, NDUFAF4, MRTO4, UNG, HK2, MCM4, PA2G4, TMEM97, SRM, NOLC1
HALLMARK_MYC_TARGETS_V1	EPRS1, BUB3, EIF3D, SNRPB2, RACK1, PCBP1, SRSF7, NCBP1, GSPT1, SRSF3, SF3B3, PSMA1, PSMD1, PSMA2, PSMB2, SMARCC1, MRPS18B, CBX3, MRPL23, IMPDH2, STARD7, XPO1, DEK, G3BP1, ABCE1, EIF4A1, SNRPD1, VBP1, SLC25A3, ILF2, EIF1AX, CSTF2, SF3A1, RPS10, RAD23B, SSBP1, ORC2, EIF4G2, PSMD14, RRM1, PSMA4, TRA2B, PSMD7, DDX18, TFDP1, PSMD3, RNPS1, EIF2S1, NDUFAB1, SNRPD3, UBE2E1, SRSF2, APEX1, MCM6, TOMM70, RPS5, HNRNPR, ACP1, CCT3, CCT7, SRSF1, CNBP, HNRNPC, LSM7, ERH, USP1, CANX, RPL6, PPIA, SSB, UBA2, KARS1, XRCC6, COX5A, AP3S1, PRPF31, KPNA2, GOT2, **MYC**, PPM1G, EIF4H, EEF1B2, TARDBP, RPL22, EIF3J, NOP16, PSMA7, NCBP2, SNRPG, NME1, PABPC1, FBL, RPS6, HNRNPD, TYMS, HDAC2, SERBP1, CYC1, SYNCRIP, HNRNPU, KPNB1, TCP1, XPOT, CCT4, EIF2S2, RPS3, RPLP0, GNL3, NAP1L1, EIF4E, IARS1, PRDX3, CTPS1, RAN, NHP2, RSL1D1, TRIM28, DDX21, PHB2, **CDK4**, SET, PTGES3, ETF1, RPS2, AIMP2, VDAC1, SNRPA1, RRP9, CCNA2, CCT5, HSP90AB1, SRPK1, NPM1, NOP56, HSPE1, CDC45, RUVBL2, RPL14, PRDX4, CCT2, TUFM, MCM2, PCNA, GLO1, MCM7, SNRPA, HSPD1, MAD2L1, POLD2, PABPC4, C1QBP, EXOSC7, HNRNPA1, PRPS2, LDHA, RFC4, MCM4, PA2G4, SRM, RANBP1, ODC1, NOLC1, IFRD1, H2AZ1, CDK2
