# Supplementary material for: Long non-coding RNA GRASLND links melanoma differentiation and interferon-gamma response
Source: Front Mol Biosci. 2024 Sep 27;11:1471100. doi: 10.3389/fmolb.2024.1471100 (PMC11466874; doi:10.3389/fmolb.2024.1471100)
Supplement: Supplementary file 10 [file Table8.pdf]

Table S8: *List of upregulated genes from HALLMARK pathways obtained from GSEA of RNA sequencing results after GRASLND knockdown in 501Mel cells. Striking genes related to observed phenotypes are highlighted in red.*

**HALLMARK Pathway**	**Upregulated genes**
HALLMARK_APICAL_JUNCTION	GNAI1, IRS1, JUP, YWHAH, GNAI2, ITGA10, CDH3, ITGA9, SYK, TRO, CERCAM, FSCN1, ADAM23, MYH9, PBX2, CTNNA1, TMEM8B, ICAM1, ACTN4, B4GALT1, VASP, STX4, NECTIN2, NECTIN3, CRAT, MAPK11, ITGA3, BMP1, MSN, SKAP2, SGCE, SPEG, ITGB1, AKT3, PARVA, MAPK14, THBS3, RSU1, CTNND1, CD99, NECTIN1, IKBKG, SHC1, CD276, NLGN2, ZYX, TJP1, NF1
HALLMARK_COAGULATION	A2M, MMP14, S100A13, CTSB, GSN, LRP1, USP11, MSRB2, SPARC, CRIP2, KLF7, FURIN, TIMP1, BMP1, CPQ, TF, ANXA1
HALLMARK_EPITHELIAL_MESENCHYMAL_TRANSITION	SNTB1, FUCA1, MMP14, MATN3, LGALS1, LRP1, CD59, CCN2, TNFAIP3, SPARC, TPM4, WIPF1, COPA, TIMP1, SGCB, LAMC1, P3H1, BMP1, SERPINE2, COL6A2, ITGB5, RHOB, ITGB1, ECM1, **TGFB1**, EMP3, PCOLCE, GADD45B
HALLMARK_IL6_JAK_STAT3_SIGNALING	A2M, IL6ST, PDGFC, STAT2, IFNAR1, TNFRSF21, IL10RB, HMOX1, **STAT3**, TNFRSF1A, SOCS3, STAM2, **TGFB1**, PIM1, IRF9, TYK2, IL13RA1, ACVR1B
HALLMARK_WNT_BETA_CATENIN_SIGNALING	PTCH1, GNAI1, NKD1, PPARD, LEF1, NUMB, HDAC5, AXIN2, NCSTN, HEY1, HEY2, FRAT1
HALLMARK_COMPLEMENT	CBLB, MMP14, S100A13, DOCK9, CTSB, GNAI2, RASGRP1, LRP1, DOCK4, CD59, TNFAIP3, PLA2G4A, GNG2, CD55, CASP4, PDP1, TIMP1, STX4, CALM3, DGKH, PSEN1, CD46, LIPA, CPQ, CASP7, USP8, VCPIP1, PIM1, CDK5R1, DGKG, USP15, EHD1, CASP9, BRPF3
HALLMARK_KRAS_SIGNALING_UP	GPRC5B, FUCA1, JUP, ETV1, TMEM158, ABCB1, TFPI, ETV5, TNFAIP3, CCSER2, SDCCAG8, RABGAP1L, HDAC9, GLRX, MAP7
HALLMARK_TNFA_SIGNALING_VIA_NFKB	**SMAD3**, PNRC1, NR4A2, TSC22D1, CEBPD, KLF9, SLC2A3, IL6ST, FOSL2, NFIL3, TNFAIP3, LITAF, BCL6, NFAT5, BCL2A1, CFLAR, ICAM1, B4GALT1, TANK, MXD1, KDM6B, DRAM1, ZFP36, SNN, PPP1R15A, SERPINB8, RHOB, PDLIM5, NFKB2, SOCS3, FJX1, PER1, DNAJB4, GADD45B, BTG1, EHD1, KLF6, TIPARP, PHLDA1, TNIP1
HALLMARK_KRAS_SIGNALING_DN	PTPRJ, NR4A2, YPEL1, CPEB3, SLC29A3, ADRA2C, MAST3, COQ8A, PDK2, MFSD6, PLAG1, IDUA, SNN, CHST2
HALLMARK_HYPOXIA	SLC2A1, PNRC1, CAV1, PAM, STC1, SLC2A3, HS3ST1, TES, SDC2, WSB1, CCN2, FOSL2, NFIL3, TNFAIP3, MT2A, NDRG1, CITED2, MYH9, P4HA2, CCNG2, KLF7, GLRX, VHL, IDS, RORA, HMOX1, NAGK, ZFP36, PPP1R15A, CHST2, B3GALT6, MAP3K1, ENO3, GBE1, ZNF292, GAA, KLHL24, PIM1, PDK1
HALLMARK_IL2_STAT5_SIGNALING	PTCH1, AHNAK, SNX9, SLC39A8, BMPR2, AHR, ABCB1, SYT11, CTLA4, SPRY4, SLC2A3, NFIL3, NDRG1, SNX14, CDC42SE2, RABGAP1L, ITGA6, FURIN, TNFRSF21, MXD1, ALCAM, RORA, SLC1A5, IFITM3, IKZF4, ITIH5, PRAF2, EOMES, TWSG1, RHOB, ENO3, SOCS2, ECM1

HALLMARK_MITOTIC_SPINDLE	KLC1, PREX1, ARL8A, KIFAP3, SORBS2, GSN, CYTH2, SPTBN1, DOCK4, PKD2, SUN2, NEDD9, CLIP2, FSCN1, DYNLL2, SPTAN1, PLEKHG2, MYH9, CAPZB, CSNK1D, NUMA1, AKAP13, ARHGAP27, ARHGAP5, HDAC6, ARFGEF1, ACTN4, ARHGAP29, RASA2, CLIP1, RICTOR, BCAR1, CNTRL, ALS2, ABI1, CDC42, SOS1, KIF3C, PDLIM5, CD2AP, STK38L, RAB3GAP1
HALLMARK_P53_PATHWAY	FUCA1, MXD4, TSC22D1, SLC7A11, PTPN14, S100A10, ERCC5, PLXNB2, SLC35D1, NDRG1, **CDKN2B**, IP6K2, COQ8A, PITPNC1, ZFP36L1, PERP, SERTAD3, MXD1, PRMT2, PPM1D, BLCAP, DRAM1, EPHA2, HMOX1, TSPYL2, PPP1R15A, CSRNP2, RALGDS, TXNIP, HEXIM1, TGFB1, ABCC5, CDK5R1, TRIB3, JAG2, BTG1, APAF1, RAB40C, DDB2, MKNK2, DEF6
HALLMARK_HEME_METABOLISM	RANBP10, SLC2A1, ATP6V0A1, SLC7A11, CTSB, SIDT2, EZH1, LRP10, LMO2, OPTN, TMCC2, NARF, P4HA2, BCAM, CAT, AGPAT4, ENDOD1, YPEL5, TMEM9B, ELL2, MARK3, KHNYN, PSMD9, LPIN2, NEK7, ACP5, CIR1, EIF2AK1, ARL2BP, RBM5, ADD1, SEC14L1, ALDH6A1, MGST3
