# Supplementary material for: Long non-coding RNA GRASLND links melanoma differentiation and interferon-gamma response
Source: Front Mol Biosci. 2024 Sep 27;11:1471100. doi: 10.3389/fmolb.2024.1471100 (PMC11466874; doi:10.3389/fmolb.2024.1471100)
Supplement: Supplementary file 11 [file Image1.pdf]

Supplementary Material

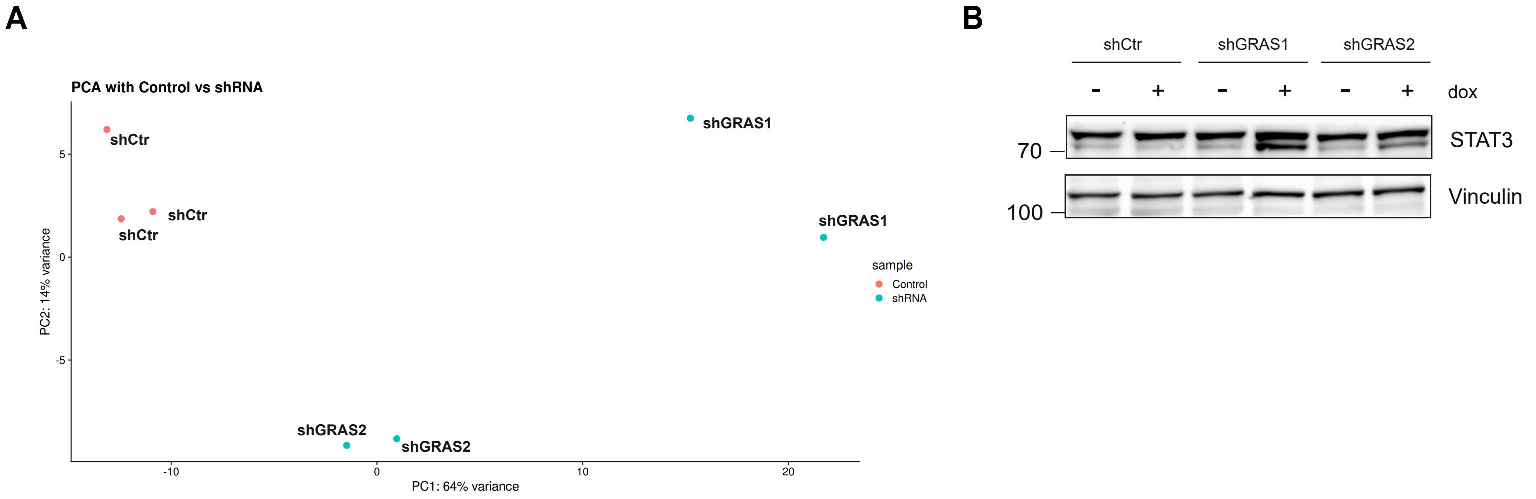

Figure S1. GRASLND knockdown induces melanoma phenotype switching **(A)** PCA plot of RNA sequencing samples. Red dots show three replicates of shCtr samples, blue dots are shGRAS1 and shGRAS2 samples, with 2 replicates each. **(B)** Expression of STAT3 after GRASLND knockdown determined by western blotting. Vinculin, loading control. Representative data of two independent experiments.
